# Supplementary material for: Eye blinks synchronize with musical beats during music listening
Source: PLoS Biol. 2025 Nov 18;23(11):e3003456. doi: 10.1371/journal.pbio.3003456 (PMC12626317; doi:10.1371/journal.pbio.3003456)
Supplement: S1 Text — (DOCX) [file pbio.3003456.s001.docx]

**Supporting Information**

**S1 Text. Differences between original and reverse music versions.**

We quantified the acoustic differences between the original and reverse versions of musical pieces. As shown in Fig 1C, the temporal modulation patterns were identical across versions, reflecting the preserved beat structure. Pulse clarity was also preserved, as only the order of beats was reversed, not the waveform itself. Regarding phrasal structure, our previous study using the same stimuli showed that neural tracking of phrasal structure remained robust even in the reverse versions [1], indicating that listeners continued to perceive regular 8-beat phrases. Although the cadence—the final beat of a phrase—became the initial beat in the reversed version, its harmonic and rhythmic salience allowed it to retain its function as a phrasal boundary, albeit with a temporal shift. The tonal center also remained unchanged. Given that the temporal structure, beat regularity, and phrasal organization were preserved across conditions, we infer that attentional engagement and temporal prediction processes (i.e., what and when expectations) were comparable between the two versions. This may explain why listeners rated both versions similarly favorable (Fig 1D).

To further assess acoustic and tonal differences, we extracted a set of low-level (dynamic complexity, sensory dissonance, pitch salience, average loudness, root mean square), tonal (key, scale, chord change rate) and rhythm (beats per minute, danceability) descriptors using Essentia 2.1[2]. As shown in S1 Fig, only the chord change rate (S1D Fig)—a key contributor to harmonic progression familiarity—differed significantly between conditions (*t*_(9)_ = 2.836, *p* = 0.020, Cohen’s *d* = 0.897). All other descriptors showed no significant differences. Key and scale were identical between conditions for each piece (S1 Table).

**References**

1. Teng X, Larrouy-Maestri P, Poeppel D. Segmenting and Predicting Musical Phrase Structure Exploits Neural Gain Modulation and Phase Precession. J Neurosci. 2024:e1331232024.

2. Bogdanov D, Wack N, Gómez E, Gulati S, Herrera P, Mayor O, et al. Essentia: An audio analysis library for music information retrieval. ISMIR2013. p. 493-8.
