# Supplementary figures and images for: Eye blinks synchronize with musical beats during music listening
Source: PLoS Biol. 2025 Nov 18;23(11):e3003456. doi: 10.1371/journal.pbio.3003456 (PMC12626317; doi:10.1371/journal.pbio.3003456)

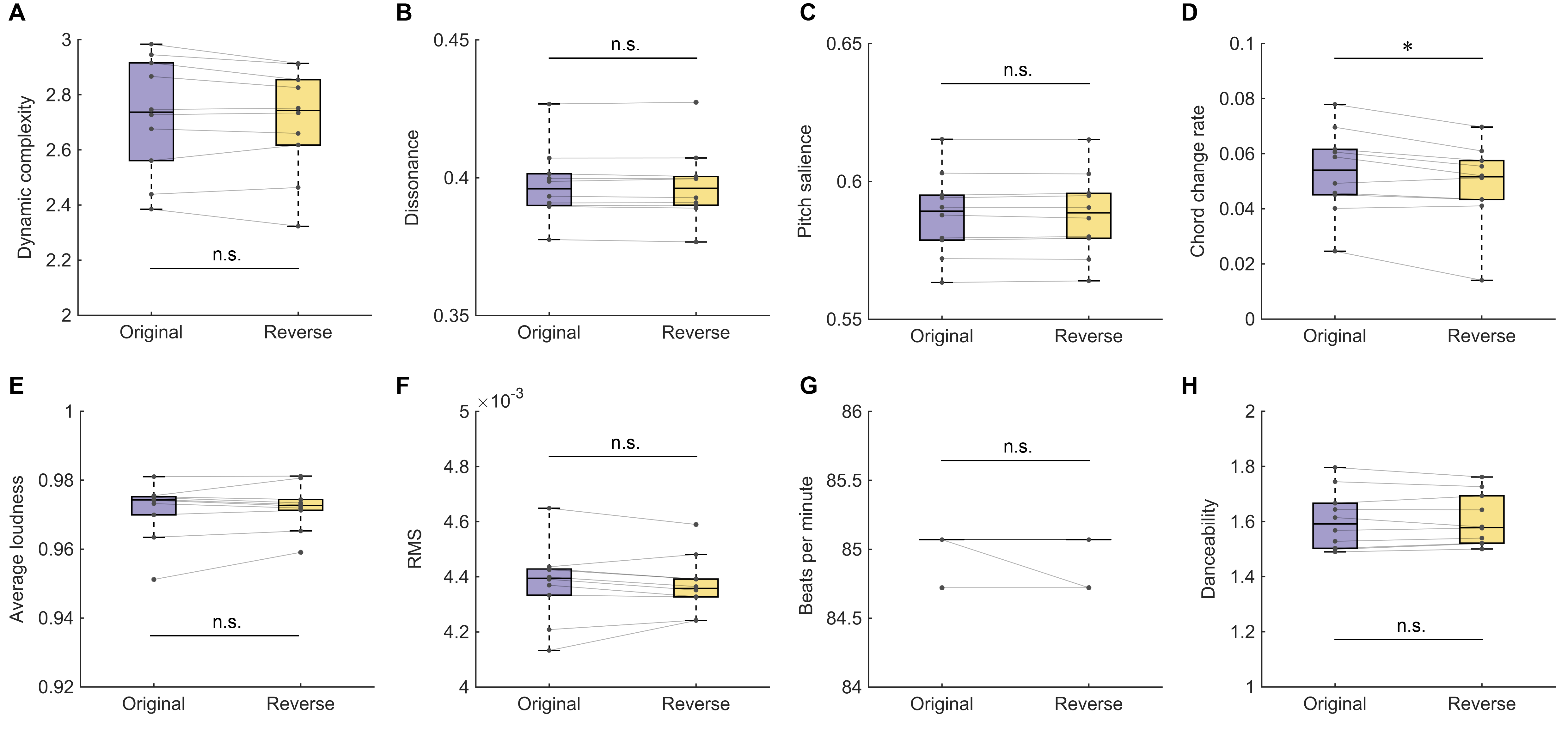

Supplement: S1 Fig — * p < 0.05; n.s., not significant. (TIF) [file pbio.3003456.s002.tif]

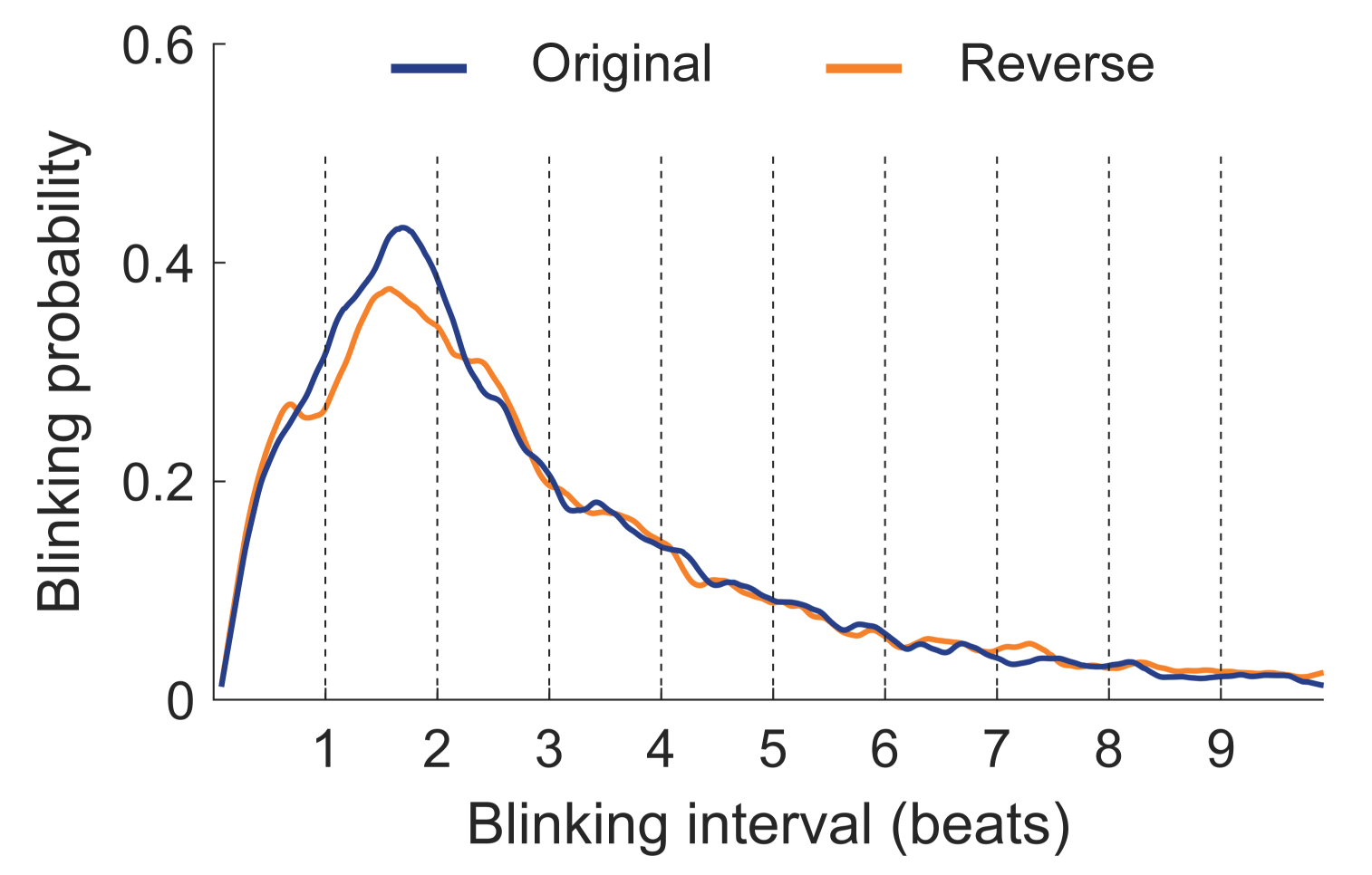

Supplement: S2 Fig — The length of each musical beat is 0.706 s. The figure displays that the inter-blink interval is most often one to two beats, consistent with what we observe in Fig 2. (TIF) [file pbio.3003456.s003.tif]

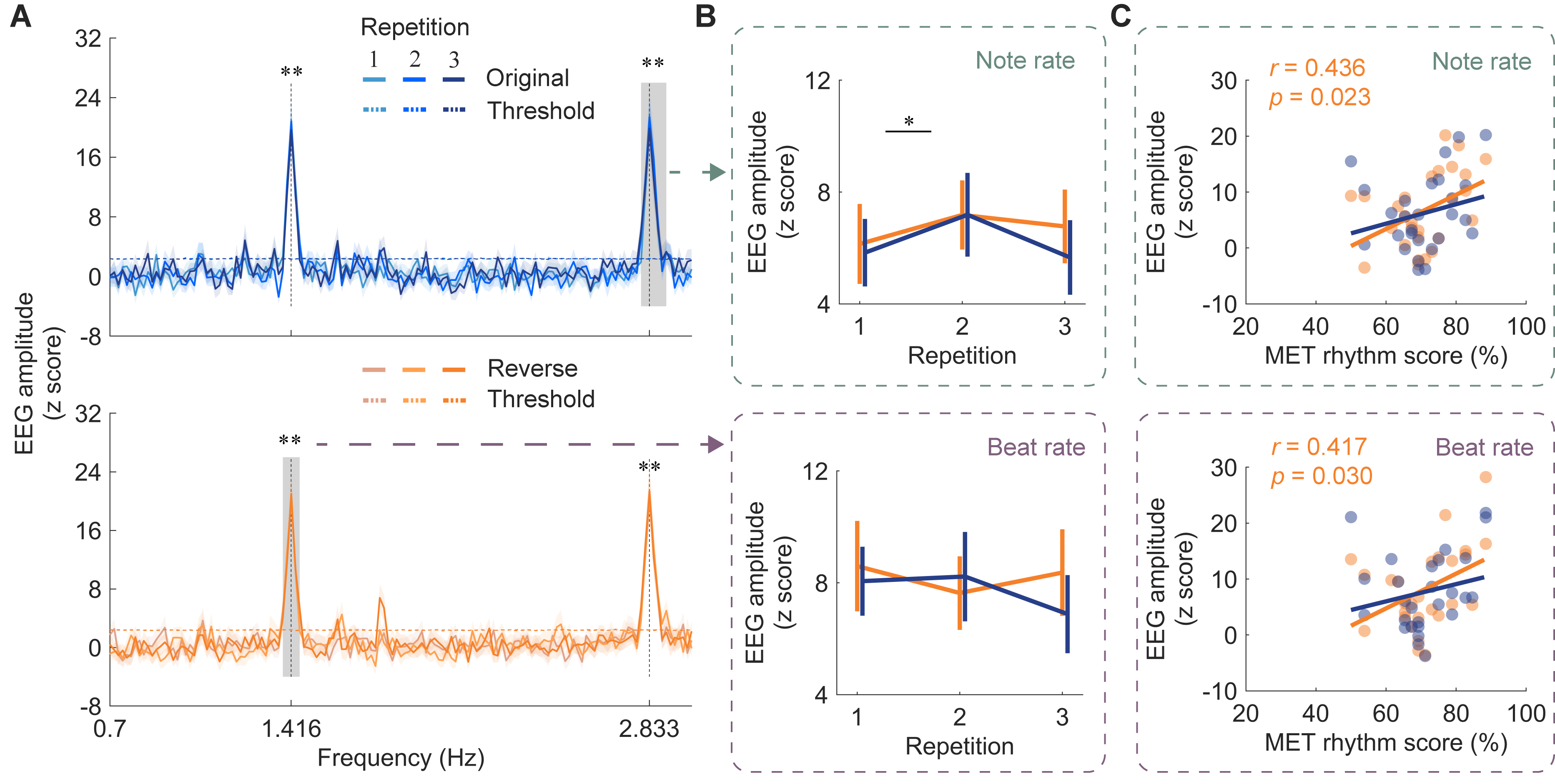

Supplement: S3 Fig — (A) Neural response spectra for two versions consistent with Fig 2D. The colored boxes indicate the frequency ranges where the amplitude was above the threshold. (B) EEG amplitude at note and beat rates. Error bars denote 1 SEM across participants. Note-rate EEG response increased with repeated exposure of music. (C) Correlation between the musical ear test (MET) rhythm score and EEG amplitude at note and beat rates. The two EEG responses were both correlated with the MET rhythm score for the reverse version. Colored dots indicate individuals. * p < 0.05, ** p < 0.01. (TIF) [file pbio.3003456.s004.tif]

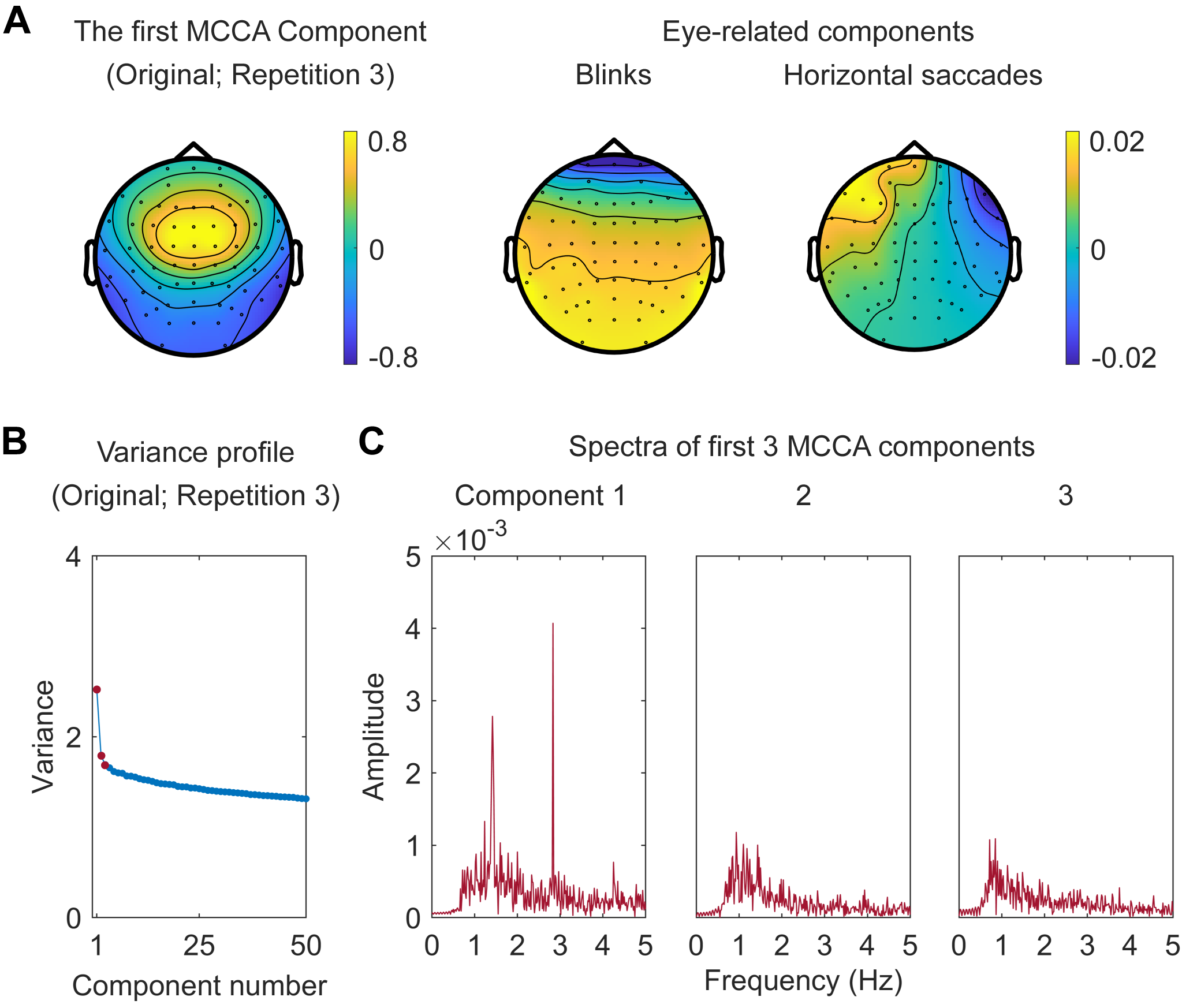

Supplement: S4 Fig — (A) Contrasting scalp topographies of the first MCCA component during the third presentation of the original condition (left) and eye-related components extracted using independent component analysis (ICA) (right), highlighting clear spatial dissociation between neural and ocular sources. (B) Variance of summary components (SCs) extracted from MCCA. The variance of the first SC explains a considerable amount of variance. (C) Amplitude spectra of first three MCCA components for the third presentation in the original condition. The spectrum of the first component shows amplitude peaks corresponding to the beat rate and note rate (the first harmonic of the beat rate), further supporting the first MCCA component, but not other components, contained the neural signals induced by beat and note structures in the music pieces. Similar results are shown for other repetitions and other reversal conditions. (TIF) [file pbio.3003456.s005.tif]

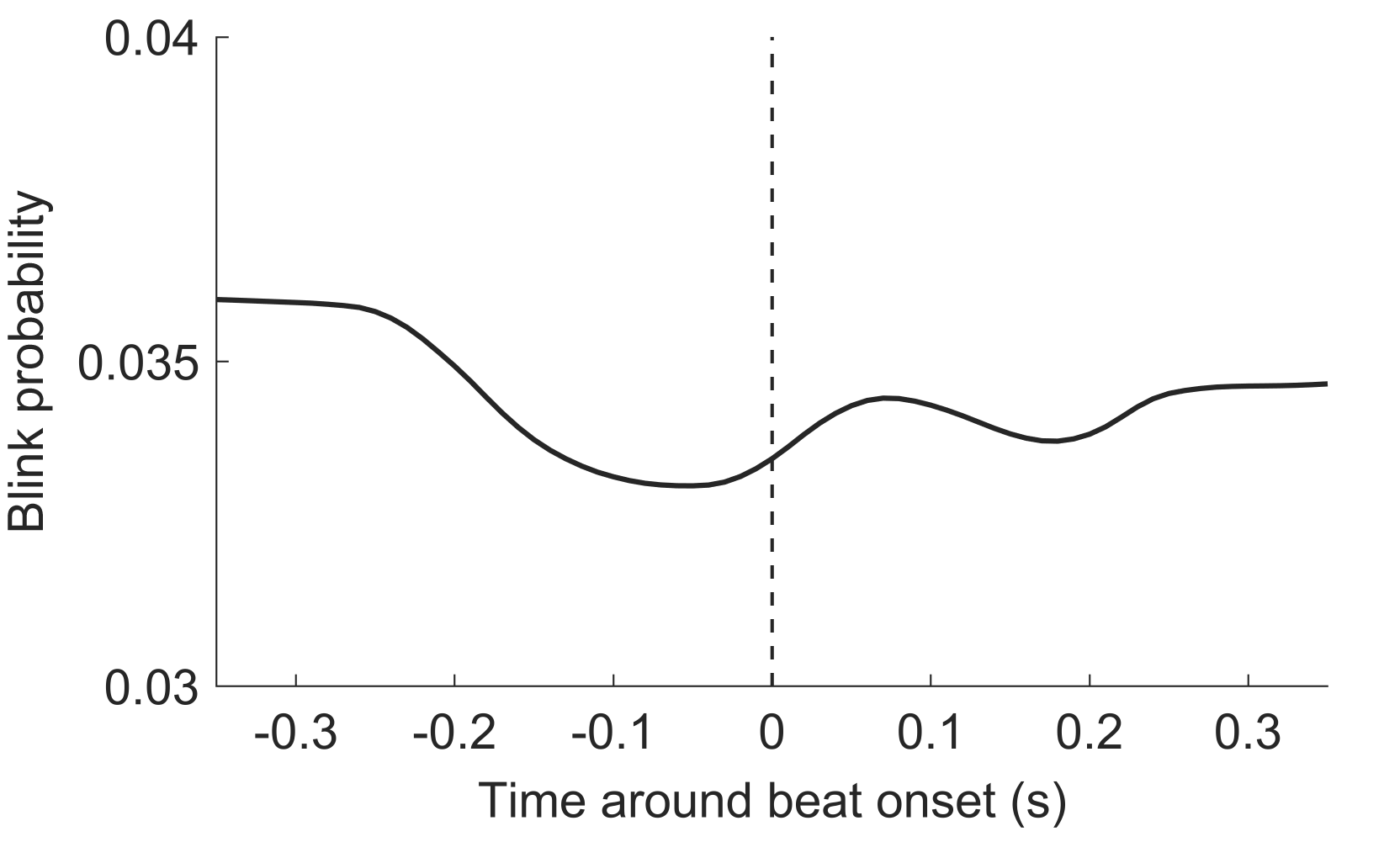

Supplement: S5 Fig — Blinks were more likely to occur between two musical beats. The vertical dashed line represents beat onset. N = 32 participants. (TIF) [file pbio.3003456.s006.tif]

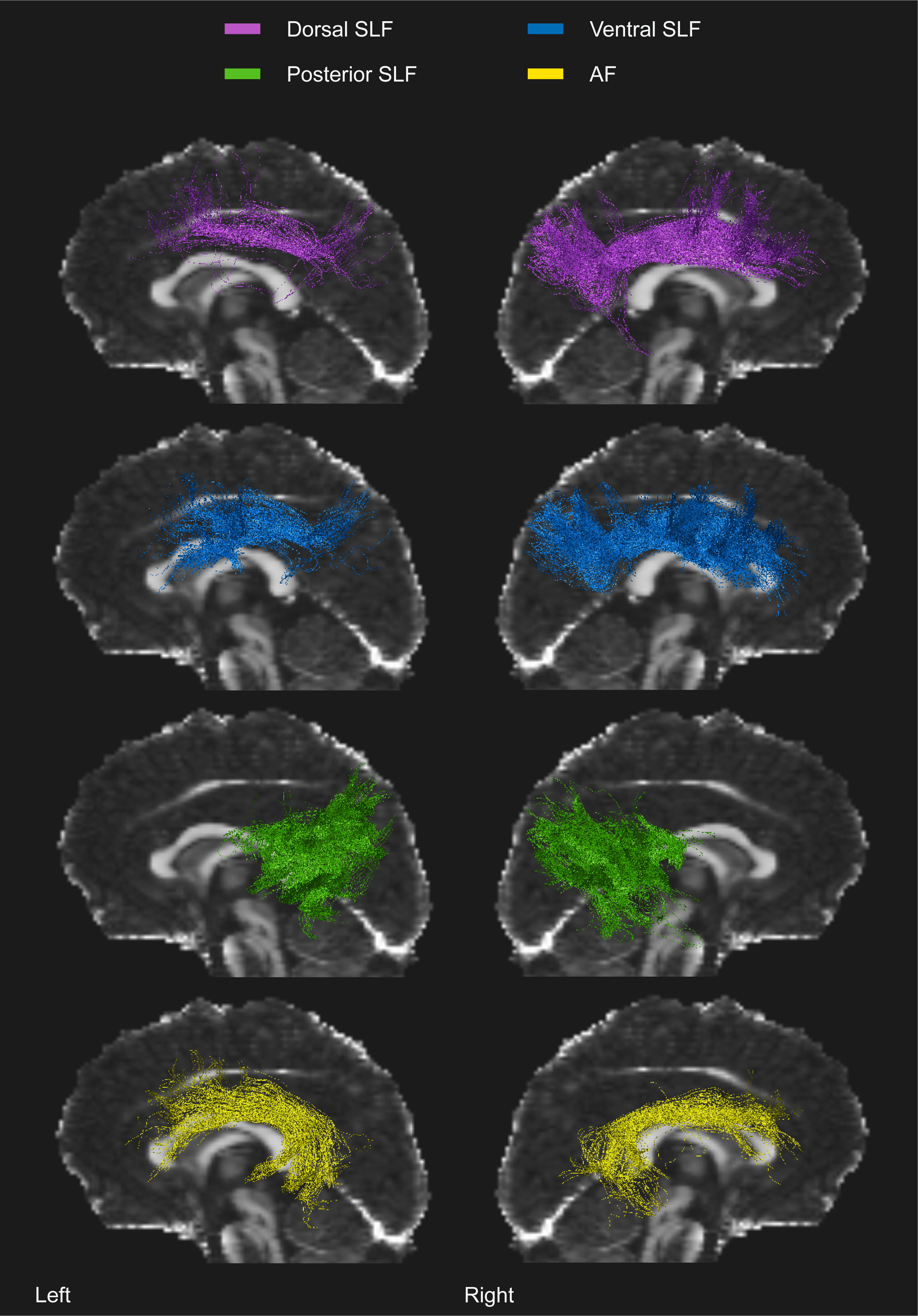

Supplement: S6 Fig — Dorsal, ventral and posterior segments of SLF are depicted in purple, blue and green. The AF is depicted in yellow. (TIF) [file pbio.3003456.s007.tif]
