# Supplementary material for: Eye blinks synchronize with musical beats during music listening
Source: PLoS Biol. 2025 Nov 18;23(11):e3003456. doi: 10.1371/journal.pbio.3003456 (PMC12626317; doi:10.1371/journal.pbio.3003456)
Supplement: S1 Table — (DOCX) [file pbio.3003456.s008.docx]

**S1 Table. Comparisons of key and scale between the original and reverse versions of musical pieces.**

| Key/scale | | | | | | | | | | |
| --- | --- | --- | --- | --- | --- | --- | --- | --- | --- | --- |
| Piece number | 1 | 2 | 3 | 4 | 5 | 6 | 7 | 8 | 9 | 10 |
| Original | A minor | A major | G major | G minor | D major | E major | Bb major | G minor | F major | D minor |
| Reverse | A minor | A major | G major | G minor | D major | E major | Bb major | G minor | F major | D minor |
