# Supplementary material for: Eye blinks synchronize with musical beats during music listening
Source: PLoS Biol. 2025 Nov 18;23(11):e3003456. doi: 10.1371/journal.pbio.3003456 (PMC12626317; doi:10.1371/journal.pbio.3003456)
Supplement: S2 Table — Values are r (p). P was estimated by partial correlation adjusting for the musical rhythm ability. * Bonferroni-corrected p < 0.05. (DOCX) [file pbio.3003456.s009.docx]

|  |  | Microstructural index | | | | | Laterality index | | | | | |
| --- | --- | --- | --- | --- | --- | --- | --- | --- | --- | --- | --- | --- |
|  |  | FA | NDI | | ODI | | FA | | NDI | | ODI | |
| Blink synchronization | L dorsal SLF | 0.044  (0.839) | -0.307  (0.144) | | -0.234  (0.272) | | 0.149  (0.488) | | 0.065  (0.763) | | -0.181  (0.398) | |
|  | R dorsal SLF | -0.126 (0.558) | -0.342  (0.102) | | -0.075  (0.726) | |  |  |  |  |  |  |
|  | L posterior SLF | -0.444  (0.240) | **-0.540**  **(0.048) *** | | 0.189 (0.377) | | -0.187  (0.381) | | 0.078  (0.716) | | 0.260 (0.220) | |
|  | R posterior SLF | -0.220  (0.302) | -0.490 (0.120) | | -0.039  (0.858) | |  |  |  |  |  |  |
|  | L ventral SLF | -0.190  (0.373) | -0.401  (0.052) | | 0.085  (0.691) | | -0.174  (0.417) | | -0.128  (0.551) | | 0.227  (0.286) | |
|  | R ventral SLF | -0.024  (0.911) | -0.315  (0.134) | | -0.210  (0.324) | |  |  |  |  |  |  |
|  | L AF | -0.318  (0.130) | -0.500  (0.104) | | -0.145  (0.499) | | 0.128  (0.552) | | 0.243  (0.253) | | -0.091  (0.671) | |
|  | R AF | -0.275  (0.194) | -0.524  (0.072) | | -0.005  (0.981) | |  | |  | |  | |
| Neural entrainment | L dorsal SLF | 0.031  (0.885) | -0.266  (0.209) | -0.269  (0.204) | | 0.498  (0.104) | | 0.245  (0.249) | | -0.400  (0.053) | |  |
|  | R dorsal SLF | -0.486  (0.128) | -0.411  (0.368) | 0.187  (0.383) | |  | |  | |  | |  |
|  | L posterior SLF | -0.504  (0.096) | -0.503  (0.096) | 0.321  (0.126) | | -0.362  (0.082) | | -0.136  (0.525) | | 0.404  (0.200) | |  |
|  | R posterior SLF | -0.147  (0.493) | -0.334  (0.111) | -0.040  (0.851) | |  | |  | |  | |  |
|  | L ventral SLF | -0.321  (0.126) | -0.526  (0.064) | 0.050  (0.816) | | -0.198  (0.355) | | -0.379  (0.068) | | 0.097  (0.651) | |  |
|  | R ventral SLF | -0.234  (0.271) | -0.280  (0.186) | -0.061  (0.776) | |  | |  | |  | |  |
|  | L AF | -0.272  (0.199) | **-0.560**  **(0.032) *** | -0.189  (0.376) | | 0.222  (0.298) | | 0.128  (0.551) | | -0.026  (0.906) | |  |
|  | R AF | -0.321  (0.126) | -0.515  (0.080) | -0.102  (0.636) | |  | |  | |  | |  |
