# Supplementary material for: Eye blinks synchronize with musical beats during music listening
Source: PLoS Biol. 2025 Nov 18;23(11):e3003456. doi: 10.1371/journal.pbio.3003456 (PMC12626317; doi:10.1371/journal.pbio.3003456)
Supplement: S3 Table — Values are mean ± SD (units: blinks/min). bpm, beats per minute. (DOCX) [file pbio.3003456.s010.docx]

| **Experiment 1** | | | | |
| --- | --- | --- | --- | --- |
|  | Repetition 1  (1.416 Hz) | Repetition 2  (1.416 Hz) | Repetition 3  (1.416 Hz) | |
| Original | 19.99 ± 10.37 | 20.02 ± 10.98 | 19.99 ± 11.24 | |
| Reverse | 19.21 ± 9.59 | 19.00 ± 9.75 | 19.17± 9.33 | |
| **Experiment 2** | | | | |
|  | 66 bpm  (1.100 Hz) | 85 bpm  (1.416 Hz) | 120 bpm  (2 Hz) |  |
| Original pieces | 19.76 ± 13.47 | 20.58 ± 15.74 | 19.37 ± 13.73 |  |
| Tone sequences | 18.73 ± 12.92 | 19.96 ± 13.63 | 18.42 ± 13.16 |  |
| Baseline | 13.43 ± 11.50 | | |  |
| **Experiment 3** | | | |  |
|  | 85 bpm  (1.416 Hz) | | | |
| Auditory target detection | 21.32 ± 12.88 | | |  |
| **Experiment 4** | | | |  |
|  | 85 bpm  (1.416 Hz) | | | |
| Visual target detection | 15.18 ± 9.59 | | |  |
