# Supplementary material for: Eye blinks synchronize with musical beats during music listening
Source: PLoS Biol. 2025 Nov 18;23(11):e3003456. doi: 10.1371/journal.pbio.3003456 (PMC12626317; doi:10.1371/journal.pbio.3003456)
Supplement: Attachment — Submitted filename: Response to Reviewers_R1.docx [file pbio.3003456.s013.docx]

We thank the editor and three reviewers for your constructive feedback. In response to your thoughtful suggestions, we have thoroughly revised the manuscript. Below we provide point-by-point responses to the concerns and questions raised. We believe these revisions have significantly improved the manuscript, and we hope all issues have been addressed satisfactorily. Thank you for the opportunity to resubmit our work.

**Reviewer #1**: The present paper investigates oculomotor activity, in particular eye blinks, while participants listen to music or sequences of tones - either attentively listening or performing a visual detection task. In addition, the authors measured EEG activity in one of the experiments and performed a link with acquired white matter data. The findings are discussed in relation to active sensing and dynamic attending frameworks.

The present study is timely and integrates in recent work using eye-movement measurements in listening tasks.

**Response:** Thank you for your summary and positive comments.

While overall sound, I have the following concerns about the present manuscript, including its presentation. The nested, interlaced presentation of the 4 experiments is difficult to follow and is missing numerous information (see below). It would certainly be easier to follow - and also more complete - to change to a more classical presentation with methods/results/discussion for each of the experiments.
**Response:** Thank you for your advice. We try to follow the format recommended by PLOS Biology, which organizes the manuscript into Introduction, Result, Discussion and Methods sections. We understand the reviewer’s concern regarding the readability of the nested presentation of the four experiments. To improve clarity, we have **specified which experiment each part of the results refers to in the Results section** and **revised the Methods section to explicitly link each methodological component to its corresponding experiment**. For instance, the “Experimental design and procedure” section has been divided into 4 subsections corresponding to each experiment. Similarly, the data acquisition and preprocessing has been reorganized into “Blink acquisition and preprocessing (Experiment 1-4)”, “EEG acquisition and preprocessing (Experiment 1)”, and “DWI acquisition and preprocessing (Experiment 1)”. In addition, we have clearly indicated the corresponding experiment in each analysis description to facilitate comprehension.

The integration of this work in previous recent research should be further developed, such as Jin et al. (2018). While briefly cited in the discussion, it should be further presented already in the introduction as this study shows how eye activity including blinks follow temporal regularity in speech and isochronous tone sequences. The here submitted study is thus not the first addressing this tracking possibility with blinks and this should be acknowledged from the start.

**Response:** Thank you for the suggestion. We were aware of this study, and have revised our manuscript so that Jin et al. (2018) was discussed adequately and make clear to readers that relevant studies on eye activities and hearing exist in the Introduction.

Line 69-72: “Research on sequence processing has demonstrated that eye blinks can track temporal regularities of both linguistic structures in speech and non-linguistic structures in isochronous tone and visual sequences [29], reflecting temporal attention.”

Please note that Jin et al.’s work addresses event segmentation in speech streams and the chunking of non-linguistic sequences, which differs in focus from our study’s emphasis on tracking musical beats.

It remains nevertheless an interesting, novel study using music together with other measures.

**Response:** Thank you for the positive evaluation.

The study presentation needs to further integrate in recent work on pupillometry, also showing the tracking of temporal regularity (e.g., Fink et al., 2018; 2024; Spiech et al., 2024) as do free eye movements (Schubert et al., 2025; Gehmacher et al., 2024). Discussion of these various measures together with the here acquired blink, EEG and anatomical data would provide a more complete picture for the reader and bring further our understanding of eye-behavior as a window into perception and cognition.
**Response:** Thank you for the references and helpful suggestion. We have incorporated findings on pupillary dynamics in the Discussion section to provide a more comprehensive perspective on the relationship between eye-behavior and musical perception.

Line 483-493: “In addition to eye blinks, prior studies have demonstrated that pupil size is modulated by microtiming asynchronies between musical beats [52], linking pupil dynamics to temporal attention and specific note configurations [53, 54], and more recently, directly to musical beat structure [30]. In other auditory contexts, both eye blinks and pupil size have been shown to track hierarchical linguistic or non-linguistic structures, reflecting a modulation of temporal attention [29]. Future research should investigate whether blink synchronization extends to higher-level musical structures, such as meter or phrase, and elucidate the relationship between blink activity and pupil dynamics. This helps to clarify the underlying cognitive processes and mechanisms involved, and differentiate oculomotor synchronization in music from that in speech processing.”

And we have already contrasted the present results of Experiment 4 with prior gaze studies, and highlighted the critical importance of integrating pupillometry, eye blinks and other oculomotor measures in the limitation part.

Line 547-551: “A prior study has demonstrated that gaze aligns with speech dynamics and the strength is modulated by selective attention [63]. In our task, participants were instructed to attend to visual stimuli, which may have inhibited the processing of auditory information and contributed to the absence of blink synchronization.”

Line 608-611: “Furthermore, given that pupil dynamics [30, 52-54] and saccades [31] are found to be implicated in processing auditory rhythms and musical structures, the coordination between blinks, pupil size and saccades in beat perception warrants further investigation to advance our understanding of eye movements in music listening.”

Material.
The authors used musical material in its original form, but also a reversed form. It is surprising that participants liked both versions equally well, with harmonic progressions and melodic/rhythmic features being altered. The reverse version reversed the "order of beats in each original piece". The authors should further analyze the created differences between original and reversed versions, notably in how far tonal and melodic structures and harmonic progressions were changed, as well as the tonal centers, pulse clarity, phrase structures etc. This seems relevant as tonal structures also guide temporal processing (e.g., what/when predictions, Jones & Boltz, 1989).

**Response:** Thank you for this thoughtful and important question. The reversed stimuli are retrogrades of the original Bach chorales, which are highly regular with isochronous beat patterns, without any syncopation or irregular irreversible temporal patterns that would make retrograde presentation problematic. In fact, in classical composition, retrograde techniques (Permutatio), such as those found in the *Crab Canon*, were used by Bach, Mozart and other composers, demonstrating the musical validity of such manipulations. Not all musical pieces can be played backward, but due to the regularity of Bach chorales, reversing the order of beats does not disrupt the fundamental musical structures. Specifically, the phrase structures-8 beats per phrase-is preserved in the reverse version. Thus, listeners still perceive regular phrasing in both conditions.

We have quantified the acoustic differences between the two conditions (Fig 1C), in which we showed that the **temporal modulation patterns** of the original and the reverse versions were identical, as the beat structure remained unchanged. **Pulse clarity** was also preserved as we reversed only the order of beats, not the waveform itself. Regarding the **phrase structures**, as shown in our previous study [1], neural tracking of phrasal structure remained robust even in the reverse versions, supporting the idea that listeners still perceive 8-beat phrases. Regarding the **cadence**, while the ending beat of a phrase in the original version became the first beat of the reverse version, it continued to function as cadential marker due to its harmonic and rhythmic salience. Thus, the functional boundaries of phrases were maintained, albeit with a temporal shift. In addition, the **tonal center** was unchanged by the reversal. Lastly, your reference to Jones & Boltz is highly relevant. Since the temporal structure, beat regularity and phrasal structure remain intact across conditions, we believe that the attention and prediction (what and when expectations) processes should be comparable between two versions. This may explain why listeners rated both versions equally favorably. Another factor is that although to a trained ear, those pieces are different, to naïve listeners or who without much exposure to Bach chorales, both versions likely appear as well-structured and coherent musical pieces.

Furthermore, to address your concern more directly, we have extracted a set of low-level (dynamic complexity, sensory dissonance and pitch salience) and tonal descriptors (key, scale and chord change rate in the progression) for all musical stimuli using Essentia 2.1 [2]. As shown in S1 Fig, **chord change rate** (D)—the primary determinant of harmonic progression familiarity—demonstrated a significant difference between conditions, whereas all other descriptors, including **dynamic complexity (A), sensory dissonance (B) and pitch salience (C)**, did not differ between conditions. Moreover, as shown in S1 Table, **key** and **scale** were identical between conditions for each piece.

These results support the interpretation that while harmonic progression is affected by reversal, most other perceptual and structural features remain comparable.

We have added that information in Supporting Information (see S1 Text):

“**Differences between original and reverse music versions**

We quantified the acoustic differences between the original and reverse versions of musical pieces. As shown in Fig 1C, the temporal modulation patterns were identical across versions, reflecting the preserved beat structure. Pulse clarity was also preserved, as only the order of beats was reversed, not the waveform itself. Regarding phrasal structure, our previous study using the same stimuli showed that neural tracking of phrasal structure remained robust even in the reverse versions [1], indicating that listeners continued to perceive regular 8-beat phrases. Although the cadence—the final beat of a phrase—became the initial beat in the reversed version, its harmonic and rhythmic salience allowed it to retain its function as a phrasal boundary, albeit with a temporal shift. The tonal center also remained unchanged. Given that the temporal structure, beat regularity, and phrasal organization were preserved across conditions, we infer that attentional engagement and temporal prediction processes (i.e., what and when expectations) were comparable between the two versions. This may explain why listeners rated both versions similarly favorable (Fig 1D).

To further assess acoustic and tonal differences, we extracted a set of low-level (dynamic complexity, sensory dissonance, pitch salience) and tonal (key, scale, chord change rate) descriptors using Essentia 2.1 [2]. As shown in S1 Fig, only the chord change rate (S1D Fig)—a key contributor to harmonic progression familiarity—differed significantly between conditions (*t*_(9)_ = 2.836, *p* = 0.020, Cohen’s *d* = 0.897). All other descriptors, including dynamic complexity (S1A Fig), sensory dissonance (S1B Fig), and pitch salience (S1C Fig), showed no significant differences. Key and scale were identical between conditions for each piece (S1 Table).”

**S1 Fig. Comparisons of dynamic complexity (A), sensory dissonance (B), pitch salience (C) and chord change rate (D) between the original and reverse versions of musical pieces.**

| Key/scale | | | | | | | | | | |
| --- | --- | --- | --- | --- | --- | --- | --- | --- | --- | --- |
| Piece number | 1 | 2 | 3 | 4 | 5 | 6 | 7 | 8 | 9 | 10 |
| Original | A minor | A major | G major | G minor | D major | E major | Bb major | G minor | F major | D minor |
| Reverse | A minor | A major | G major | G minor | D major | E major | Bb major | G minor | F major | D minor |

**S1 Table. Comparisons of key and scale between the original and reverse versions of musical pieces.**

The authors adapted the original musical scores, for example eliminating rhythmic or acoustic cues for phrasal structures". The material is created and exported with MuseScore software. This software might include expressive performance cues (related to timing and intensity). The authors should confirm that they removed all these cues to create mechanical versions.

**Response:** We have ensured that all expressive performance cues—such as variations in timing and intensity—were removed from the musical materials. Specifically, all pieces were exported from MuseScore as mechanical versions, without expressive rendering. In addition, all pieces were normalized according to the average root mean square sound pressure level (SPL) of 70 dB. MuseScore generates computer-produced music sounds, which is highly regular without any temporal jittering, exactly what we need for systematically studying musical beat perception and its relationship with eye blinks without introducing performance-related confounds.

To further confirm the absence of expressive variation, as suggested by the reviewer, we extracted a set of low-level descriptors (average loudness, spectral rms) and rhythm descriptors (bpm, danceability) for all pieces using Essentia 2.1. As shown below, all metrics revealed non-significant between-condition differences, confirming that all musical materials were equivalent in expressive performance characteristics.

For each experiment, the auditory material should be made available to the readers as supplementary materials.

**Response:** According to the reviewer’s suggestion, we have uploaded all musical materials along with preprocessed data to the OSF folder: <https://osf.io/xhfn4/>. This has been shown in Data availability statement.

The reversed versions are designed as "unfamiliar control". However, when presented three times in a row, the item is not unfamiliar anymore. Can the authors further explain the rational of their design?
**Response:** We apologize for the confusion regarding the term “unfamiliar control”. What we intended to convey is that the reversed versions were designed to control for **long-term familiarity**. The original versions of Bach chorales and their typical harmonic progressions are likely familiar to many listeners due to prior exposure, even if infrequent. This familiarity could influence how listeners may process those stimuli, potentially engaging memory-related neural mechanisms in addition to perceptual ones.

In contrast, the reversed versions were newly created for this experiment and not naturally occurring musical forms. Participants would not have encountered them prior to the study, making them unfamiliar in terms of **long-term memory**. This distinction allows us to better isolate the effects of prior musical exposure on neural and behavioral responses.

Nevertheless, the reviewer is right that with 3 repetitions in the experiment, a degree of **short-term familiarity** may develop. However, this type of familiarity is part of what we are able to measure and model directly through our neural and behavioral analyses. Importantly, given the complexity and lack of musical convention in the reversed versions, it is unlikely that participants could form strong or detailed memory representations of these stimuli after only three repetitions.

We have revised the sentence in Lines 108-109:

“This design ensured that the reverse versions served as controls for long-term familiarity.”

Line 532: "from 22.59 s to 59.30s" - why were musical pieces with this large range of durations used? Entrainment to the beat might differ between shorter and longer pieces. Different trial lengths also lead to different number of data points over the pieces, thus more or less stable "average amplitude spectra".

**Response:** We did not deliberately control the duration of the musical pieces but simply selected pieces with more than four musical phrases and with regular beat and phrasal patterns. As a results, the durations varied naturally, reflecting the diversity found in real-world music. Our goal was to prioritize ecological validity by using naturalistic musical stimuli rather than artificially constructed sequences.

We fully acknowledge the potential impact of varying piece lengths on beat tracking and amplitude spectra estimation. To address this, we took several steps in our analysis pipeline to mitigate the influence of duration differences. Specifically, as now clarified in the Methods section:

Line 812-822: “The blink data for each trial and participant were transformed into the frequency domain using a FFT with zero-padding of 6000 points. To account for differences in trial length, we conducted surrogate testing in the spectral domain to derive a null distribution of the amplitude modulation spectra for each independent variable. We then z-scored the raw amplitude spectra with respect to the surrogate mean, effectively normalizing them to remove biases introduced by varying stimulus durations. Next, we averaged the amplitude spectra across the 10 pieces for each condition. To identify robust blink and neural tracking of musical structures, we identified the lower and upper bounds of the significant frequency ranges and extracted average spectral amplitudes within these frequency ranges for further statistical analyses.”

Additionally, while piece durations varied, all stimuli were long enough to contain multiple beat cycles—sufficient for inducing beat entrainment, as supported by prior studies that have reliably measured beat tracking using even shorter musical segments. We further minimized the impact of duration differences by averaging the modulation spectra across all pieces within each condition, ensuring stable and comparable estimates.

In summary, although exact piece lengths were not matched, we implemented appropriate normalization and averaging procedures to control for their potential effects. This approach reflects a balance between experimental control and ecological relevance, which we believe strengthens the generalizability of our findings.

Further, line 575: The rest period is of a duration of 3min. Why was this duration chosen, being different from the music durations?

**Response**: Thank you for raising this important point. The 3-minute rest duration was chosen for two reasons. First, it allowed us to obtain a sufficient baseline for blink activity, ensuring adequate temporal coverage to capture natural blink patterns and establish a stable baseline. Second, the duration aligns with our frequency-domain analysis requirements. Specifically, it provided the 6000 data points required for the FFT analysis, enabling consistent comparison with the task-related data. To enhance the signal-to-noise ratio, the 3-min baseline was partitioned into three 60-second epochs during analysis. This approach allowed us to preserve both data quality and analytic consistency

We have clarified this in the revised sentence in Lines 697-699:

“Before the main experiment, participants underwent a 3-min rest period while looking at a blank screen to establish a stable baseline for blink activity.”

Line 543: "we extract twenty-four melodic sequences" … consisted of eight chords". Please clarify the material description as chords contain at least two notes played simultaneously, thus differing from melodic sequences.

**Response:** Sorry for the mistake and confusion. The description has been modified as:

Line 662-664: “In Experiment 3, the original versions of ten musical pieces used in Experiment 1 were divided into twenty-four discrete segments, each comprising two complete phrases. Each phrase consisted of eight beats.”

Line 564: The figure refers to repetitions of the same piece. Clarify "in a randomized order" here to avoid misunderstanding.

**Response:** We apologize for the misunderstanding. We have rephrased the sentence as:

Line 684-685: “In each block, participants listened to each of the 5 pieces three times in a row, and the piece order was randomized for each participant.”

Line 571 "melody condition and rhythm condition" - does melody condition refer to the full original version (melody+harmony+rhythm) of Experiment 1? Please clarify labels.

**Response:** Yes, the melody condition refers to the original version used in Experiment 1. To avoid potential confusion, we have revised the wording in this section to align with the terminology used in the Results section.

Line 656-658: “In Experiment 2, the original pieces were selected from eight of the ten musical pieces used in Experiment 1, and the tone sequences consisted of frequent standard tones (440 Hz) with timing structures identical to the original pieces.”

Line 694-697: “Different types of stimuli (original pieces or tone sequences) were presented in separate blocks, with the types alternating every 5 trials. Stimuli were presented to participants according to a balanced Latin square design for every type, and the order of stimulus types was counterbalanced across participants.”

Line 577: participants press the space bar as soon as possible. How does programming and realizing a motor response influence subsequent blinks?

**Response:** While motor responses could theoretically influence subsequent blinks, our design effectively minimized such confounding effect. Participants were instructed to press the key only when detecting timbre deviants, which were exclusively inserted in two additional musical pieces not used in the main analyses. This task ensured sustained attentional focus on the auditory stimuli. Crucially, the two trials containing timbre deviations were excluded from all subsequent analyses. We have clarified these points in both the Methods and Results sections:

Line 351: “Trials containing timbre deviants were excluded from analyses.”

Line 658-661: “To ensure participants’ attention remained focused, we randomly inserted one to three chords deviating in timbre (guitar) into the remaining two musical pieces. These two pieces were interspersed within the block but excluded from further analyses.”

Line 580: "participants were presented with two versions of the twenty-four melodic sequences" - What were these two versions? Original/reversed or rhythmic? Please clarify.

**Response:** Here, the different versions corresponded to varying positions of the pitch deviants within the same musical segment. We have clarified this point in the Methods section.

Line 667-668: “Each segment was manipulated into two versions with the pitch deviant inserted at different positions.”

Line 705-708: “Participants were presented with two versions of the twenty-four auditory segments, each presented twice, leading to 96 segments in total (24 segments × 2 versions × 2 times). Stimuli were presented in a pseudorandomized order to ensure that no segment or version was repeated consecutively.”

Line 588: "used as the original versions in Experiment 1" - please clarify.

**Response:** Sorry for the confusion. We have revised the description to enhance methodological clarity.

Line 715-717: “Participants engaged in a visual target detection task while listening to ten musical pieces—the same original versions used in Experiment 1. Each musical piece was presented twice in a pseudorandomized order.”

Line 593: "each musical piece contained at least one target and up to three for each condition with the number of targets per condition was the same" - Please clarify which are the conditions and also how it is possible to have the same number of targets per condition in each piece when it varies from one to three.
**Response:** The experimental conditions refer to: (1) the **on-beat condition**: the visual target presented in synchrony with the musical beat onset, and (2) the **off-beat condition**: the visual target presented out-of-synchrony with the musical beat onset. These two conditions were specified in the Methods section:

Line 718-720: “The visual target appeared either synchronously with the beat onset (on-beat condition) or randomly at one of nine temporal positions between two beats (off-beat condition).”

We apologize for the confusion in the original phrasing. The total number of visual targets per piece varied from one to four, depending on the piece’s duration. Specifically, shorter musical pieces contained fewer targets, while longer pieces incorporated more. However, within each individual piece, the number of targets was matched across the two conditions. For instance, if a piece contained one on-beat target, it also contained one off-beat target. Clarification has been modified as:

Line 721-723: “1) each musical piece contained at least one and up to four visual targets per condition, depending on the duration of piece, with an equal number of on-beat and off-beat targets within each piece.”

Data analyses:
-    The blink rate section (line 752ff) refers to 85 beats per minute. How were the other tempi analyzed? The window here is described as -0.353 to 0.353s.

**Response:** The blink rate (which has been renamed as blink probability in the revised manuscript) metric was analyzed only in Experiment 4, which utilized musical stimuli with a fixed tempo of 85 beats per minute. We have explicitly shown that this analysis only applied for Experiment 4 in the revised version (Lines 897-902):

“**Blink probability (Experiment 4)**

In this analysis, we derived blink time series within a window from -0.353 to 0.353 s in relative to the beat onset, determined by the musical tempo (85 beats per minute). Blink probability at each time point was defined as the average value across trials for each participant, quantifying the likelihood of blink occurrence within the defined time window. Results are illustrated in S5 Fig.”

-    EEG data might be affected by blink occurrence and frequency. The authors comment on this (page 10) but should further explain this and provide the related data in the results section.

**Response:** Thank you for this important comment. To maintain focus in the main manuscript, we presented the relevant analysis and results in S4 Fig and its figure caption, with a brief description of the result in the main text.

Lines 224-228: “Given the distinct scalp topographies of the first MCCA neural component and the eye-related components (including blinks and horizontal saccades) extracted via ICA (S4A Fig), which demonstrate clear spatial dissociation between neural and ocular sources, the observed blink-neural relationship cannot simply be attributed to blink artifacts.”

**S4 Fig. Multiway canonical correlation analysis (MCCA).** **(A)** Contrasting scalp topographies of the first MCCA component during the third presentation of the original condition (left) and eye-related components extracted using independent component analysis (ICA) (right), highlighting clear spatial dissociation between neural and ocular sources. **(B)** Variance of summary components (SCs) extracted from MCCA. The variance of the first SC explains a considerable amount of variance. **(C)** Amplitude spectra of first three MCCA components for the third presentation in the original condition. The spectrum of the first component shows amplitude peaks corresponding to the beat rate and note rate (the first harmonic of the beat rate), further supporting the first MCCA component, but not other components, contained the neural signals induced by beat and note structures in the music pieces. Similar results are shown for other repetitions and other reversal conditions.

-    Does task-related movement influence blink activity? If yes, how is this corrected?

**Response:** Task-related movement did not confound the analysis of blink activity in the present study.

Specifically, in Experiment 1, participants only pressed a key once—after the third presentation of each musical piece—to rate their liking. In Experiment 2, as delineated above, key presses were required only when detecting timbre deviants, which were exclusively inserted in two additional musical pieces that were excluded from subsequent analyses. In Experiment 3 and 4, participants performed auditory or visual target detection tasks, but targets were presented only from the second musical phrase onward. For analyses, we focused on the time window spanning from 1 s after musical piece onset until the first target appearance, during which participants maintained fixation on the central cross on the screen and no motor response was required.

This design ensured that the blink data were recorded during a movement-free interval. Moreover, prior studies have demonstrated that reliable blink tracking responses can be observed within similarly timed segments of tone sequences or speech stimuli [3].

Line 636: Add a reference justifying these limits.

**Response:** Thank you for the suggestion. We have added the reference.

Lines 751-752: “Blink durations shorter than 50 ms or longer than 500 ms were excluded from the analysis [78].”

Line 639: "data's mean" - is this the mean over trials in one participant or the group?

**Response**: Thank you for pointing it out. The term ‘data’s mean’ refers to the mean across trials within each participant. We have clarified it:

Lines 754-756: “Blinks with durations exceeding three standard deviations from the participant-specific mean across trials, and trials with blink rates exceeding three times the participant-specific standard deviation, were removed.”

Line 650: MCCA: add some information presenting this method.

**Response**: A brief introduction of MCCA has been presented in the Results section:

Lines 192-196: “…we applied multiway canonical correlation analysis (MCCA) to extract components linked to music listening (see Methods). This approach enabled the extraction of auditory neural signals specific to music listening and facilitated the analysis of single-trial data for each musical piece and repetition.”

Line 774-778: “Finally, we concatenated all trials into one matrix to derive the most common component of the EEG signals reflecting neural responses to musical pieces, using MCCA [80]. The first MCCA component was projected back to each participant and each trial, and used for subsequent analysis.”

Line 681: "all independent variables" - please clarify which ones.

**Response:** All independent variables are blink amplitudes in Experiments 1-4 and EEG amplitudes in Experiment 1. We have rephrased this sentence in Lines 819-822:

“To identify robust blink and neural tracking of musical structures, we identified the lower and upper bounds of the significant frequency ranges and extracted average spectral amplitudes within these frequency ranges for further statistical analyses.”

Line 744: Did the authors have hypotheses for laterality asymmetry?
**Response:** We have elaborated our hypotheses about the DTI results and especially the laterality asymmetry in the Results section:

Lines 303-311: “Previous studies have found a positive correlation between fractional anisotropy (FA) of the left (but not right) arcuate fasciculus (AF) and auditory-motor synchronization [46-48]. In addition to the AF, the superior longitudinal fasciculus (SLF), which connects auditory and dorsal premotor regions through parietal regions, has also been implicated in auditory-motor synchronization and beat perception [18]. Based on these findings, we hypothesized that individuals with better blink synchronization or neural entrainment would exhibit stronger structural connectivity in the left AF and SLF, or greater left-lateralization of these tracts.”

Results:
The authors performed a median split which seems to require excluding 5 participants (Figure 4A). In light of Figure 4's distribution, one might wonder whether median split is appropriate here. Indeed, the median split approach with ANOVAs has been criticized (see, e.g., MacCallum, Zhang, Preacher, & Rucker, 2002, DOI: 10.1037//1082-989X.7.1.19). Including a continuous music background variable, e.g., as a continuous covariate in an AN(C)OVA, might be more appropriate.

**Response:** Thank you for the valuable suggestion. Indeed, given the limited sample size and relatively homogeneous distribution of blink amplitude in our cohort, median split is deemed unsuitable for this study. Following the suggestions here, we implemented partial correlation analysis with musical rhythmic ability (measured by the MET rhythm score) as a covariate to directly examine the associations between blink synchronization and microstructural metrics of white matter tracts. We have revised our manuscript to describe these methodological refinements:

Lines 320-327 in the Results section: “Partial correlation analyses controlling for musical rhythmic ability revealed a significant negative correlation between blink amplitude and the NDI of the left posterior SLF (pSLF) (*r*_(22)_ = -0.540, *p* = 0.048, Bonferroni-corrected, Fig 4A). Neural entrainment was also significantly and negatively correlated with the NDI of the left AF (*r*_(22)_ = -0.560, *p* = 0.032, Bonferroni-corrected, Fig 4B). These findings suggest that better blink and neural synchronization may be associated with lower neurite density in the left pSLF and AF. No significant correlations were found for other microstructural indices (see S2 Table for full statistics).”

**Fig 4. Partial correlations between blink/neural synchronization and white matter microstructure, controlling for musical rhythmic ability. (A)** Blink amplitude averaged across the original and reserve versions was negatively correlated with the neurite density index (NDI) of the left posterior superior longitudinal fasciculus (pSLF). **(B)** EEG amplitude at the beat rate averaged across the two versions was negatively correlated with the NDI of the left arcuate fasciculus (AF). Colored dots represent individuals.

Lines 920-923 in the Methods section: “Correlations between blink synchronization and neural entrainment (Fig 2E), as well as between blink/neural synchronization and white matter microstructural properties (Fig 4), were analysed using partial correlations controlling for the MET rhythm score, with multiple comparisons corrected by the Bonferroni correction method.”

We have also updated this finding in the abstract and discussed this finding in the Discussion section:

Abstract: “Blink synchronization performance was linked to white matter microstructure variation in the left superior longitudinal fasciculus, a key sensorimotor pathway.”

Lines 457-458: “On a structural level, we found that the microstructural connectivity of the left pSLF played an important role in the strength of blink synchronization (Fig 4).”

Lines 508-527: “Moreover, we found that the microstructural integrity, indexed by NDI, of the left pSLF served as the structural basis for blink synchronization. The pSLF connects the auditory cortex and inferior parietal lobule encompassing the intraparietal sulcus (IPS), where the parietal eye field (PEF) is located [55]. Lower NDI, which reflects reduced neurite density, was associated with better blink synchronization performance (Fig 4A). Notably, NDI derived from the NODDI model provides a more precise and sensitive estimate of neurite morphology than FA [56]. A series of studies have similarly reported that lower neurite density in white and gray matter is linked to higher intelligence, better reading and phonological skills, and improved cognitive performance [57-59]. Consistent with these findings, our results suggest that lower NDI in the left pSLF supports higher auditory-motor synchrony ability, potentially reflecting greater neural efficiency associated with synaptic pruning. However, given the relatively small sample size, replication and stability assessment in larger cohorts are necessary. Furthermore, we did not find significant associations between blink synchronization and the structural properties of the dSLF, which connects the PEF to the dorsolateral frontal area encompassing the frontal eye field (FEF). This null finding may stem from limited statistical power due to the modest sample size and/or high homogeneity of musical ability within our participant group. Future studies incorporating larger and more diverse populations with a broader range of musical expertise will be essential for fully addressing this question. ”

Blink synchronization decreased with tempo, leading to non-significant findings for 120 bpm. How do the authors explain this? Might it be related to the fact that 500ms inter-beat-interval is too short regarding potential recovery periods?

**Response:** Thank you for the insightful questions. We have already discussed these points in the Discussion section. This paragraph elucidates the disappeared blink synchronization at 120 bpm for musical pieces based on stimulus characteristics, specifically tempo and acoustic features.

Line 528-538: “Notably, we did not find significant blink synchronization at a tempo of 120 beats per minute for the musical stimuli (Fig 5C). This implies a rate limit for blink synchronization, a phenomenon also observed in finger tapping and covert synchronization studies [50, 60]. Given differentially preferred tempo of rhythmic behaviours among motor effectors [49], further research is required to determine the optimal tempo range for robust blink synchronization to musical beats. Interestingly, blink synchronization was restored when original musical pieces were replaced with tone sequences at 120 beats per minute. This result is consistent with a recent study showing that repeated acoustic units facilitate auditory-motor synchronization in a subgroup of the population [61], inspiring us to take acoustic features into consideration in future experiments.”

The tempo of 120 bpm corresponds to a beat rate of 2 Hz. Empirical evidence from prior research has robustly demonstrated that a response peak at 2 Hz was observed for the blink activity during both auditory and visual sequence perception [3]. Consequently, we conclude that the recovery period is unlikely to constitute a significant confounding factor in the current study.

Line 252: "Firstly ..." - which experiment is concerned here?
**Response:** It refers to Experiment 1. We have added this specification at the beginning of this section:

Line 300-303: “In Experiment 1, we examined the relationship between white matter microstructural properties and blink synchronization/ neural entrainment performance to further explore the structural variability associated with auditory-motor synchronization.”

Participants:
Musical background is indicated with the Gold MSI output. Please explain to the readers what level of musical expertise is reflected by these numbers, and complete this information by the more usual description of years of musical training/instruction.

**Response:** The Goldsmiths Musical Sophistication Index (GOLD-MSI) is a self-report inventory which includes six dimensions: active music engagement, self-reported perceptual abilities, musical training, self-reported singing abilities, and sophisticated emotional engagement with music. The musical training subscale provides a comprehensive assessment of life history of formal musical training. It contains 7 items such as “I have had 0 / 0.5 / 1 / 2 / 3-5 / 6-9 / 10 or more years of formal training on a musical instrument (including voice) during my lifetime”, with all items scoring on the 7-point scale.

To evaluate not only the years of musical training but also the amount of training of participants, we selectively employed the musical training subscale, on a scale from 7: no training to 49: more training than 99% of the random sample (n=147,633).

Relevant clarification has been added:

Line 634-639: “The “musical training” subscale of the Goldsmiths Musical Sophistication Index (Gold-MSI) questionnaire [77] was used to evaluate the amount of musical training of participants, on a scale from 7: no training to 49: more training than 99% of the random sample. All participants scored an average of 17.45 (SD = ± 7.60, range: 7 - 39) on the musical training subscale and none of them was musician.”

Discussion
Line 408: "motor cortical oscillations … actively predicts impending blink activation" - the wording must be turned down or completed with additional analyses.
Line 410: "the brain continuously updates its predictions" - also here, complete with the appropriate analyses over time.

**Response:** Thank you for raising this thoughtful concern. This interpretation is based on our temporal response function analysis, which revealed a time-locked neural oscillatory response gradually preceding blink onset across the first to third repetitions (Fig 3D and 3E), suggesting a refinement of neural prediction for upcoming blinks through short-term exposure.

This result has been reported in Lines 287-290: “It also showed a significant main effect of repetition (*F*_(2,54)_ = 4.799, *p* = 0.012, *η_p_^2^* = 0.151), with higher weights during the third presentation than the first presentation (*p* = 0.004, Bonferroni-corrected), indicating a gradual formation of prediction for blink onset in the brain.”

However, we fully acknowledge that the limited spatial resolution of EEG prevents us from conclusively localizing this activity to motor cortices. In response to your comments, we have clarified the temporal aspect of the analyses and moderated our interpretation in the manuscript as follows:

Lines 496-506: “Specifically, eye blinks were represented in cortical responses, with a time-locked neural response gradually preceding blink onset **across the first to third repetitions** (Fig 3D and 3E), suggesting **a** **refinement of neural prediction for upcoming blinks through short-term exposure**. Aligning with frameworks such as predictive coding [16, 17], active sensing [14, 15], and the ASAP hypothesis [18], our findings suggest that cortical oscillations, **potentially originating from dorsal auditory-motor pathways,** not only synchronize with incoming musical beats but **may also** **contribute to the predictive timing of blink activity**. By comparing the expected and actual timing of blinks **across repetitions**, **the brain may continuously update its temporal predictions**, leading to improved synchronization between blinks and neural signals.”

Line 460: Discuss differences in materials and tasks in these studies to better understand previous findings and the present findings.

**Response:** Thank you for the suggestion. We have expanded the paragraph to further discuss the discrepancies between these studies:

Line 567-579: “This disparity is likely attributable to several factors. First, most studies supporting rhythmic effects employed simple geometric shapes or objects as stimuli, with tasks requiring the detection or discrimination of low-level perceptual features, unlike the pseudowords used in studies yielding null findings. Second, the temporal relationship between beats and off-beat targets significantly influences outcomes. While off-beat targets in many prior studies appeared at fixed intervals with respect to the beat, in both the current study and previous studies reporting null-effects, off-beat targets were randomly distributed relative to the beat. Finally, methodological differences in experimental design may have contributed to the inconsistent findings. Studies reporting rhythmic effects typically used blocked designs, whereas our study employed an intermixed design in which on-beat and off-beat targets occurred within the same block. This design likely weakened the temporal expectations induced by the musical rhythm, thereby diminishing the rhythmic effect on target detection.”

Other points:
Line 375: "we then replicated this blink synchronization phenomenon across a broader range of tempi (66-120 bpm)…." VERSUS line 427: "we did not find significant blink synchronization at a tempo of 120 beats" - Please clarify results and their discussion.

**Response:** We apologize for this confusion. Regarding the tempo of 120 bpm, blink synchronization was absent exclusively for the original musical pieces. In contrast, blink synchronization re-emerged for the tone sequences, which constituted the central focus of the subsequent discussion. We have refined the former paragraph's wording to delineate these points more clearly.

Line 459-462: “We then replicated this blink synchronization phenomenon across a broader range of tempi (66 and 85 beats per minute) of the original musical pieces and all three tempi (66/85/120 beats per minute) in tone sequences without melodic cues (Fig 5), affirming its robustness.”

Line 289: indicate the interval between beats in msec for readers unfamiliar with bpm.

**Response:** Thank you for the thoughtful suggestion. We have added the interval between beats for each tempo in seconds, to maintain consistency with the time unit used throughout the manuscript.

Line 344-346: “we compared blink synchronization to both music and static tone sequences across three tempi (66, 85, and 120 beats per minute, corresponding to inter-beat intervals of 0.909 s, 0.706 s and 0.5 s, respectively).”

Line 293: "Similar to Experiment 1, …….. detecting a timbre deviant" - please clarify if this task is new for Experiment 2 or clarify this task for Experiment 1.
**Response:** Sorry for the confusion. This detection task is new for Experiment 2, and we have improved the relevant descriptions by removing ambiguous sentence “Similar to Experiment 1” to enhance clarity.

**Reference**

1.	Teng X, Larrouy-Maestri P, Poeppel D. Segmenting and Predicting Musical Phrase Structure Exploits Neural Gain Modulation and Phase Precession. J Neurosci. 2024:e1331232024.

2.	Bogdanov D, Wack N, Gómez E, Gulati S, Herrera P, Mayor O, et al. Essentia: An audio analysis library for music information retrieval. ISMIR2013. p. 493-8.

3.	Jin P, Zou J, Zhou T, Ding N. Eye activity tracks task-relevant structures during speech and auditory sequence perception. Nat Commun. 2018;9(1):5374.

**Reviewer #2**: This study investigates the association between the spontaneous eye-blinks and musical beat using eye-tracking, EEG recordings, white matter structural imaging, and behavioral analysis. The results showed synchrony between blink and the beat of music in original and reverse music versions. The analysis in the EEG recordings showed a relation between blink timing and neural beat tracking and differences were observed in the microstructure of the left arcuate fasciculus between low and high synchronizers. Finally, the authors established an association between blink synchronization and pitch deviant detection.

This is a large study combining eye-tracking, EEG recordings, white matter structural imaging, and behavioral analysis in order to quantify how spontaneous eye blinks synchronize with musical beats. The largest weakness of the papers lies in the fact that different subjects were used in the different experiments and that there is a lack of integration of the methodologies within a conceptual framework that integrates the observations into a hypothesis of the role of eye-blinking in beat perception. The study is descriptive in nature and does not establish a possible mechanism between audiomotor and eyeblinks for music beat perception and synchronization.
**Response:** We thank the reviewer for this insightful comment and suggestion. We are particularly grateful for this specific point, which motivates us to deepen our understanding of the underlying mechanisms and their conceptual significance.

Integrating multiple studies into a single, coherent conceptual framework is challenging—especially when presenting a newly demonstrated phenomenon. To our knowledge, spontaneous blink synchronization with musical beats has not been previously explored. We therefore conducted a thorough review of prior work to ground our findings conceptually, proposed a novel active-sensing/predictive-coding framework, and designed four experiments to test it.

Each experiment targeted different aspects to help understand the behavior:

Experiment 1: Demonstration of blink–beat synchronization

Experiment 2: Dependence on temporal versus melodic cues

Experiment 3: Behavioral relevance of synchronization

Experiment 4: Task dependence of the effect

We agree that embedding our experiments within a cohesive, mechanistic framework strengthens the manuscript. However, formulating such a framework for a novel finding often requires multiple empirical studies to elucidate underlying processes, which is the aim of our current work. While mature research typically rests on established frameworks, pioneering discoveries invite further exploration and theorizing. We hope the reviewer appreciates our effort to explore this phenomenon and our proposed frameworks as a foundation for future studies.

In our framework, spontaneous blinks are involuntary motor actions that reflect temporal predictions of upcoming beats, mediated by dorsal auditory-motor pathways and basal ganglia–premotor–oculomotor circuits. The convergence of behavioral synchronization, EEG entrainment, and white-matter microstructure (left pSLF/AF) provides initial mechanistic evidence linking blink timing to neural and structural substrates of beat prediction, rather than serving as a purely descriptive measure.

We have indeed discussed our findings with proposed frameworks:

Lines 496-507: “Specifically, eye blinks were represented in cortical responses, with a time-locked neural response gradually preceding blink onset across the first to third repetitions (Fig 3D and 3E), suggesting a refinement of neural prediction for upcoming blinks through short-term exposure. Aligning with frameworks such as predictive coding [16, 17], active sensing [14, 15], and the ASAP hypothesis [18], our findings suggest that cortical oscillations, potentially originating from dorsal auditory-motor pathways, not only synchronize with incoming musical beats but may also contribute to the predictive timing of blink activity. By comparing the expected and actual timing of blinks across repetitions, the brain may continuously update its temporal predictions, leading to improved synchronization between blinks and neural signals. This enhanced synchronization may, in turn, facilitate more efficient auditory beat processing.”

Lines 618-621: “By linking active sensing and ASAP frameworks to blink synchronization, we gain deeper insights into the coordination of auditory, motor, and visual systems, thereby enriching our knowledge of cross-modal interaction and embodied musical perception.”

Regarding subject cohorts, our rationale was twofold: first, using the same stimuli across different groups confirms that the observed blink-beat synchronization behavior replicates robustly; second, it prevents adaptation, learning effects, or familiarity with the stimuli from biasing our results. We acknowledge that a within-subject design would allow more direct comparisons, but in this case, repeated exposure could confound our findings. Science advances once at a time, and after demonstrating robustness across cohorts, we plan to conduct within-subject experiments to examine correlations across measures in future studies.

Minor comments.

Nothing is said in the introduction about the interaction between visuomotor rhythmic behavior, especially using rhythmic moving stimuli and its possible link with eye blinks.
**Response:** Thank you for the suggestion. We have revised our manuscript so that only the one previous study on eye blinks in response to periodic visual sequence is mentioned in the Introduction.

Line 69-72: “Research on sequence processing has demonstrated that eye blinks can track temporal regularities of both linguistic structures in speech and non-linguistic structures in isochronous tone and visual sequences [29], reflecting temporal attention.”

On Figure 2A, it is difficult to see which repetition of the original and reverse conditions are above the threshold. In Figure 2B is not evident whether the blink amplitude is above random, especially for the third repetition, and the question is why.

**Response:** We acknowledge that the original figure was insufficiently clear. We have adjusted line width and transparency level, and marked repetitions with statistically significant peaks above the thresholds using different colors of asterisks.

Regarding Figure 2B, the corrected amplitudes were derived within the significant frequency range (the lower bound and the upper bound of the significant frequencies among all the three repetitions), which spanned from 1.416 Hz to 1.433 Hz. We have clarified this in the Methods section:

Line 814-822: “To account for differences in trial length, we conducted surrogate testing in the spectral domain to derive a null distribution of the amplitude modulation spectra for each independent variable. We then z-scored the raw amplitude spectra with respect to the surrogate mean, effectively normalizing them to remove biases introduced by varying stimulus durations. Next, we averaged the amplitude spectra across the 10 pieces for each condition. To identify robust blink and neural tracking of musical structures, we identified the lower and upper bounds of the significant frequency ranges and extracted average spectral amplitudes within these frequency ranges for further statistical analyses.”

There are inconsistencies in the effect of the condition (original and reverse) and the repetition across figures 2 and 3 that are not easily reconcilable.

**Response:** Thank you for raising this concern. Indeed, we observed that the amplitude of blink synchronization to the beat did not significantly differ between the original and reverse conditions (Fig 2), whereas both the mutual information (MI) between blink and neural synchronization and the weights of the temporal response function (TRF) of neural oscillations in response to blinks were significantly greater in the original than reverse conditions (Fig 3). This apparent inconsistency can be explained by differences in the underlying processing mechanisms.

First, as shown in the temporal modulation patterns of music stimuli (Fig 1C), the beat structures were preserved in both the original and reverse conditions. Since blinks synchronized to the beat structure, it is reasonable that blink synchronization amplitude did not differ between conditions.

**Fig. 1C.** Modulation spectra of the stimuli showed peaks at the note and beat rates as well as their harmonics for both versions.

Second, as shown in the new S1 Fig, chord change rate (S1D Fig)—a key determinant of harmonic progression familiarity—differed significantly between the two conditions. Because both MI and TRF weights reflect the relationship between blink and neural activities, processes more closely tied to predictive timing mechanisms, the unfamiliar harmonic progression in the reverse condition likely disrupted predictive processing. This disruption, in turn, lead to diminished MI and TRF weights for the reverse condition compared to the original.

**S1 Fig.** **Comparisons of dynamic complexity (A), sensory dissonance (B), pitch salience (C) and chord change rate (D) between the original and reverse versions of musical pieces.**

We have clarified these points in the manuscript following each result:

Lines 210-214: “This suggests that although blink synchronization to the beat did not differ between the two versions due to their identical beat structures (Fig 1C), higher musical ability may be required to effectively track beat structures in the reverse version, possibly due to the unfamiliar harmonic progressions (S1D Fig).”

Lines 266-269: “This discrepancy between the two versions, despite their identical beat structures, implies that effective prediction—facilitated by the familiar harmonic progressions in the original version (S1D Fig)—may strengthen the coupling between blink and neural responses.”

The number of subjects for low and high blink synchronizers is small and the lack of differences in the two groups with their MET rhythm scores emphasizes the lack of consistency between blink behavior and rhythmic abilities in the subjects. It is not evident whether the authors used cluster correction analysis on their FA, NDI and ODI. It is difficult to associate the changes in the orientation dispersion index (ODI) of the left arcuate fasciculus in the two groups of subjects with the circuit that controls eye blinking.

**Response:** Thank you for pointing this out. Given the small sample size and relatively homogeneous musical ability across our participants, we opted against artificially dividing participants into two subgroups and instead employed partial correlation analysis to directly examine the relationship between blink synchronization and microstructural integrity. Multiple comparisons were corrected using Bonferroni correction method. New results have been added and are copied below:

Lines 320-327 in the Results section: “Partial correlation analyses controlling for musical rhythmic ability revealed a significant negative correlation between blink amplitude and the NDI of the left posterior SLF (pSLF) (*r*_(22)_ = -0.540, *p* = 0.048, Bonferroni-corrected, Fig 4A). Neural entrainment was also significantly and negatively correlated with the NDI of the left AF (*r*_(22)_ = -0.560, *p* = 0.032, Bonferroni-corrected, Fig 4B). These findings suggest that better blink and neural synchronization may be associated with lower neurite density in the left pSLF and AF. No significant correlations were found for other microstructural indices (see S2 Table for full statistics).”

**Fig 4. Partial correlations between blink/neural synchronization and white matter microstructure, controlling for musical rhythmic ability. (A)** Blink amplitude averaged across the original and reserve versions was negatively correlated with the neurite density index (NDI) of the left posterior superior longitudinal fasciculus (pSLF). **(B)** EEG amplitude at the beat rate averaged across the two versions was negatively correlated with the NDI of the left arcuate fasciculus (AF). Colored dots represent individuals.

Lines 920-923 in the Methods section: “Correlations between blink synchronization and neural entrainment (Fig 2E), as well as between blink/neural synchronization and white matter microstructural properties (Fig 4), were analysed using partial correlations controlling for the MET rhythm score, with multiple comparisons corrected by the Bonferroni correction method.”

Our updated results demonstrated that blink synchronization was significantly associated with the neurite density index (NDI) of left posterior superior longitudinal fasciculus (pSLF). This tract connects auditory regions with parietal eye field (PEF) and has been implicated in auditory-motor synchronization and beat perception. Relevant discussion has been added and is copied below:

Lines 508-527: “Moreover, we found that the microstructural integrity, indexed by NDI, of the left pSLF served as the structural basis for blink synchronization. The pSLF connects the auditory cortex and inferior parietal lobule encompassing the intraparietal sulcus (IPS), where the parietal eye field (PEF) is located [55]. Lower NDI, which reflects reduced neurite density, was associated with better blink synchronization performance (Fig 4A). Notably, NDI derived from the NODDI model provides a more precise and sensitive estimate of neurite morphology than FA [56]. A series of studies have similarly reported that lower neurite density in white and gray matter is linked to higher intelligence, better reading and phonological skills, and improved cognitive performance [57-59]. Consistent with these findings, our results suggest that lower NDI in the left pSLF supports higher auditory-motor synchrony ability, potentially reflecting greater neural efficiency associated with synaptic pruning. However, given the relatively small sample size, replication and stability assessment in larger cohorts are necessary. Furthermore, we did not find significant associations between blink synchronization and the structural properties of the dSLF, which connects the PEF to the dorsolateral frontal area encompassing the frontal eye field (FEF). This null finding may stem from limited statistical power due to the modest sample size and/or high homogeneity of musical ability within our participant group. Future studies incorporating larger and more diverse populations with a broader range of musical expertise will be essential for fully addressing this question. ”

**Reviewer #3:** The authors present 4 experiments in which they establish and replicate that blinks synchronize spontaneously to the beat structure of music, mediated by neural synchronization to the music. Blinks synchronize mainly to temporal rather than melodic properties of music, and seem to guide attention towards auditory but not visual targets. The authors further propose that individual differences in this blink-to-beat synchronization are explained by structural properties of the left arcuate fascicle. These are exciting new findings in the field of active sensing.

The study is timely, well conducted and very clearly presented. I receive it after the first revision, and obvious questions like whether the link between neural and blink synchronization is driven by spurious blink artifacts in the EEG signal have been ruled out. The perhaps weakest point in my view (although still interesting to be reported) is the trial to link blink synchronization to the microstructure of the AF. This is because low and high synchronizers do not seem to differ specifically in blink synchronization at the beat rate (S4 Fig.), but more globally. So, is it justified to call them "synchronizers" in the first place? Second, it is not clear whether this analysis was Bonferroni corrected (3 measures x 4 tracts x 2 hemispheres), but probably it was? Please clarify.

**Response:** Thank you for the positive comments and the thoughtful questions. We concur with the reviewer that the observed between-subgroup difference in blink synchronization was globally, which probably due to an artifact of artificially partitioning a homogeneous cohort. Consequently, we reanalyzed these data using partial correlation to quantify relationships between blink synchronization and microstructural properties. We acknowledge that the initial analysis omitted multiple comparisons correction; this has been rectified in the revised manuscript through implementation of Bonferroni correction method (4 tracts x 2 hemispheres) for each microstructural index. We have rewritten the relevant parts in the Results and Methods section:

Lines 320-327 in the Results: “Partial correlation analyses controlling for musical rhythmic ability revealed a significant negative correlation between blink amplitude and the NDI of the left posterior SLF (pSLF) (*r*_(22)_ = -0.540, *p* = 0.048, Bonferroni-corrected, Fig 4A). Neural entrainment was also significantly and negatively correlated with the NDI of the left AF (*r*_(22)_ = -0.560, *p* = 0.032, Bonferroni-corrected, Fig 4B). These findings suggest that better blink and neural synchronization may be associated with lower neurite density in the left pSLF and AF. No significant correlations were found for other microstructural indices (see S2 Table for full statistics).”

Lines 920-923 in the Methods: “Correlations between blink synchronization and neural entrainment (Fig 2E), as well as between blink/neural synchronization and white matter microstructural properties (Fig 4), were analysed using partial correlations controlling for the MET rhythm score, with multiple comparisons corrected by the Bonferroni correction method.”

**Fig 4. Partial correlations between blink/neural synchronization and white matter microstructure, controlling for musical rhythmic ability. (A)** Blink amplitude averaged across the original and reserve versions was negatively correlated with the neurite density index (NDI) of the left posterior superior longitudinal fasciculus (pSLF). **(B)** EEG amplitude at the beat rate averaged across the two versions was negatively correlated with the NDI of the left arcuate fasciculus (AF). Colored dots represent individuals.

Lines 507-526 in the Discussion: “Moreover, we found that the microstructural integrity, indexed by NDI, of the left pSLF served as the structural basis for blink synchronization. The pSLF connects the auditory cortex and inferior parietal lobule encompassing the intraparietal sulcus (IPS), where the parietal eye field (PEF) is located [55]. Lower NDI, which reflects reduced neurite density, was associated with better blink synchronization performance (Fig 4A). Notably, NDI derived from the NODDI model provides a more precise and sensitive estimate of neurite morphology than FA [56]. A series of studies have similarly reported that lower neurite density in white and gray matter is linked to higher intelligence, better reading and phonological skills, and improved cognitive performance [57-59]. Consistent with these findings, our results suggest that lower NDI in the left pSLF supports higher auditory-motor synchrony ability, potentially reflecting greater neural efficiency associated with synaptic pruning. However, given the relatively small sample size, replication and stability assessment in larger cohorts are necessary. Furthermore, we did not find significant associations between blink synchronization and the structural properties of the dSLF, which connects the PEF to the dorsolateral frontal area encompassing the frontal eye field (FEF). This null finding may stem from limited statistical power due to the modest sample size and/or high homogeneity of musical ability within our participant group. Future studies incorporating larger and more diverse populations with a broader range of musical expertise will be essential for fully addressing this question. ”

Overall, I believe the study is well done and needs to be presented to the community. A few minor specifications may help readers to better understand or replicate the findings, as listed below, sorted by topic.
**Response:** Thank you.

Interpretation suggestion:
-    Line 448: The authors argue that the lack of blink synchronization in Experiment 4 is because of the task irrelevance of music. Have you considered that, alternatively, participants may have withheld or more voluntarily controlled blinks (e.g., blinked less) due to the visual task? Would a tactile task, i.e., a task that diverts attention from the auditory stream but isn't visual, be an alternative to dig deeper into the reasons for the lack of effects?
**Response:** Thank you for the valuable suggestion. Employing a tactile task is an excellent idea, as it would help control for task demands while isolating the specific influence of task relevance on blink synchronization. While a tactile task was regrettably not incorporated in the current experimental design, we have explicitly acknowledged its importance in the revised Discussion section as a promising avenue for future work:

Line 548-556: “In our task, participants were instructed to attend to visual stimuli, which may have inhibited the processing of auditory information and contributed to the absence of blink synchronization. Moreover, the visual task demands may have induced strategic blink inhibition to prioritize visual perception, thereby muting the effect of music on blink synchronization. A potential ceiling effect in task performance may also have obscured active sensing mechanisms. Future studies are needed to investigate cross-modal temporal modulation of blink synchronization using more challenging tasks and modality-matched control tasks, such as tactile tasks.”

Methods specification:
-    Line 250: "First, we excluded 3 outliers" — Outliers regarding which measure? Probably blink synchronization strength, but perhaps good to spell out.

**Response:** Thank you for pointing out this. We excluded 3 outliers based on their blink response amplitudes, but the sentence describing median split methodology has been removed in the revised manuscript in accordance with our updated analytical approach.

-    Line 254: "The blink amplitude spectra for the two groups showed a significant difference around the beat frequency (S4 Fig)." — If I understood correctly, the significant beat synchronization window was between 1.416-1.433 Hz (line 129). Why do the two groups show differences in synchronization across a much broader range, from 0.7 to 2.5 Hz? How specific is the median split to blink synchronization?

**Response:** Thank you for raising this concern. As mentioned above, the median split results have been removed due to their ineffectiveness, and we conducted partial correlations to directly examine the associations between blink synchronization and white matter microstructural properties while controlling for musical rhythmic ability.

-    Line 590: What duration did the red circle have?

**Response:** We apologize for the unclear information. We have improved the description of the stimuli presentation.

Line 724-726: “The maximum duration of a red circle matched the musical beat interval (0.706 s), disappearing immediately upon spacebar response.”

-    Line 637: Was blink rate calculated based on blink onset-to-onset intervals?

**Response:** In the preprocessing stage, blink rate was quantified as the number of blinks per minute for each trial and each participant (units: blinks/min).

Lines 753-754: “Blink rate was quantified as the number of blinks per minute for each trial and participant (units: blinks/min).”

-    Line 693: "a sliding window length of 3" — Please specify the unit. Seconds? Please also specify the frequency range and duration of the Morlet wavelet.

**Response:** Thank you for the suggestion. The window length refers to cycles, and we have now specified both the units and the details about Morlet wavelet in the Methods section:

Line 831-834: “First, we extracted the time-frequency distribution of the EEG data at the beat rate (1.416 Hz) using a Morlet wavelet with a sliding window length of 3 cycles, as implemented in Fieldtrip. The analysis window spanned from 3 s before the onset to 3 s after the offset of each musical piece, with a step size of 10 ms.”

-    Line 738: "parceled by Freesurfer" — Which atlas was used and which parcels in that atlas (please provide the names of the parcels)?

**Response:** Thank you for raising the methodological point. Further clarification has been added to make the tractography procedure clearer:

Line 874-879: “Freesurfer’s automatic anatomical parcellation (aparc2009) algorithm [91] was used to define a set of 148 cortical and subcortical regions of interest (ROIs) from each individual’s anatomical image. We further divided the superior temporal gyrus into equational anterior and posterior portions, and divided the precentral gyrus into equational dorsal and ventral parts.”

-    Line 743: "mean FA, NDI and ODI values of each segment were extracted for each participant" — Maybe I overlooked it, but how were segments (tractography maps) thresholded?

**Response:** Thank you for pointing this out. We perform probabilistic tracking using iFOD2 tracking algorithm, with following parameters: 10 million streamlines for each participant, step size = 0.75, angle threshold = 45°, maximum tract length = 150 mm, fractional anisotropy cutoff = 0.1. We have supplemented these methodological details in the Methods section:

Line 863-866: “Thirdly, the whole-brain probabilistic tractography was performed to generate 10 million streamlines for each participant based on iFOD2 tracking algorithm (step size = 0.75, angle threshold = 45°, maximum tract length = 150 mm, FA cutoff = 0.1) [87].”

-    Line 752: It took me some time to understand that "blink rate" doesn't actually refer to a rate in Hz (as used in line 637), but something like a "blink probability" or "distribution around the beat". Perhaps this could be clarified.

**Response:** Yes, the reviewer is correct that the term “blink rate” here refers to the blink probability, which was defined as the proportion of blinks occurring within a specified peri-event window (-0.353 to 0.353 s in relative to the beat onset).

To prevent conceptual conflation with “blink rate” (the number of blinks per minute within each trial) used in preprocessing, we have now renamed the measure in Experiment 4 as “blink probability” – quantifying the likelihood of blink occurrence within the defined time window.

We have clarified this in the Methods section (Lines 897-902):

“**Blink probability (Experiment 4)**

In this analysis, we derived blink time series within a window from -0.353 to 0.353 s in relative to the beat onset, determined by the musical tempo (85 beats per minute). Blink probability at each time point was defined as the average value across trials for each participant, quantifying the likelihood of blink occurrence within the defined time window. Results are illustrated in S5 Fig.”

We have also revised the figure caption of S5 Fig as “**S5 Fig.** **Blink probability in relative to the beat onset in Experiment 4.** Blinks were more likely to occur between two musical beats. The vertical dashed line represents beat onset. N = 32 participants.”

-    S5 Figure and line 468-470: "Blinks were more likely to occur around the beat onset" — Well, actually it looks as if most blinks occurred 300 ms before/after the beat, i.e., exactly between two beats. Is this really compatible with the interpretation of the post-blink boost effect?

**Response:** Sorry for the confusion. Based on previous research [1], the post-blink boost effect occurs immediately after eye blinks. In the present study, blinks predominantly occurred between musical beats. When off-beat visual targets appeared, blink precisely facilitated perception of these stimuli, consequently yielding shorter reaction times in detection tasks under off-beat condition compared to on-beat condition.

While our findings aligned with the interpretation of the post-blink boost effect, our initial description of blink timing may have caused misinterpretation. We have therefore revised the characterization in the Discussion section:

Line 582-583: “As illustrated in S5 Fig, eye blinks were more likely to occur between two beats, potentially facilitating the perceptual processing of off-beat targets.”

-    Generally, 1,000 permutations are rather at the lower end of permutation testing. 5k or 10k permutations would be better.
**Response:** Thank you for the suggestion. We have reconducted the surrogate test with 5,000 permutations and replaced the statistical results and figures in our revised manuscript. Due to computational demands, however, the surrogate test for temporal response function (TRF) was still repeated 1,000 times.

Typos detected on the way:
-    Line 101: "while EEG and eye-tracking recordings simultaneously" — something is missing in this sentence.

**Response:** It has been corrected as ‘while EEG and eye-tracking data were recorded simultaneously’ (Lines 104-105).

-    Line 400: Please correct "clarity" to "clarify".

**Response:** Corrected.

-    Line 409: Please correct "synchronizes" to "synchronize".

**Response:** Corrected.

-    Line 461: Please correct "other" to "others".

**Response:** Corrected.

-    Line 495: Please correct "found to implicated" to "found to be implicated".

**Response:** Corrected.

-    Line 570: Please correct "allowed" to "were allowed".

**Response:** Corrected.

-    Line 594: Please correct "with the number of targets per condition was the same" to "... being the same" or "with the same number of targets per condition".

**Response:** It has been corrected as “1) each musical piece contained at least one and up to four visual targets per condition, depending on the duration of piece, with an equal number of on-beat and off-beat targets within each piece” (Lines 721-723).

-    Line 638: Do you mean "Blinks with durations exceeding 3 SD" rather than "Blinks with mean durations exceeding 3 SD"?

**Response:** It has been corrected as “Blinks with durations exceeding three standard deviations from the participant-specific mean across trials, and trials with blink rates exceeding three times the participant-specific standard deviation, were removed.” (Lines 754-756).

-    Line 734: Please correct "super marginal" to "supramarginal".

**Response:** Corrected.

-    Figure 1E: Does B and N on the x-axes of the power plots refer to beat-rate and note-rate? Please specify in the figure legend.

**Response:** Sorry for the omission. The legend has been modified as:

Line 127-130: “The synchronization value around the beat rate (B) was measured…Denoised signals were used to measure neural entrainment to beats (B) and notes (N).”

-    Figure 1E: Why 90 trials? Weren't these 60 trials (10 pieces x 2 versions x 3 repetitions) in Experiment 1?

**Response:** Corrected.

-    Please check the references - formatting is not always consistent.

**Response:** We have carefully checked the references to ensure consistent formatting.

**Reference**

1.	Ang JWA, Maus GW. Boosted visual performance after eye blinks. J Vis. 2020;20(10):2.
