# Supplementary material for: Eye blinks synchronize with musical beats during music listening
Source: PLoS Biol. 2025 Nov 18;23(11):e3003456. doi: 10.1371/journal.pbio.3003456 (PMC12626317; doi:10.1371/journal.pbio.3003456)
Supplement: Attachment — Submitted filename: Response to Reviewers_R3.docx [file pbio.3003456.s014.docx]

**Reviewer #1**: The revised manuscript is improved and clarified. I nevertheless have the following comments that remain, but that the authors could certainly easily respond to in a revised version.

**Response:** Thank you.

Material:

1)	In their response "To further confirm the absence of expressive variation,..", the main point is not addressed as the comment here was about expressive features in the mechanical condition, thus still having somewhat expressive basic features that the software proposed. The authors response indicates that both original and reverse conditions do not differ in expressive variations. Please clarify.

**Response:** We apologize for not fully addressing the reviewer’s point in our earlier response. To clarify: all musical materials were exported from MuseScore as strictly mechanical versions, with all expressive performance features (e.g., tempo rubato, dynamic shaping, articulation cues) disabled. MuseScore’s “playback” feature can indeed add such expressive timing and intensity variations by default, but in our stimulus preparation we explicitly selected the “no style / mechanical playback” option, thereby ensuring each note onset and intensity was uniform. Additionally, all stimuli were normalized to the same RMS sound pressure level (70 dB SPL).

Thus, the “mechanical condition” in our study did not include expressive performance cues; any residual low-level variation was due probably to the inherent score structure because of our modification of original scores rather than software-introduced expressivity. To further confirm, as reported in our first revision, we extracted low-level acoustic descriptors (average loudness, spectral RMS) and rhythmic descriptors (tempo, danceability) using Essentia 2.1, all of which showed no significant differences between original and reversed versions. These analyses support that the mechanical rendering itself contained no expressive features that could bias blink synchronization.

We have now clarified this point explicitly in the Methods section:

Line 685–687: “All stimuli were generated in MuseScore 3 and exported as wav files with a piano timbre, using the mechanical playback setting to remove expressive timing and dynamic cues. Musical excerpts were presented at a sampling rate of 44100 Hz, and their intensity level were normalized to 70 dB SPL. Low-level acoustic analyses confirmed the absence of expressive variation between conditions (S1E-H Fig).”

**S1 Fig. Comparisons of dynamic complexity (A), sensory dissonance (B), pitch salience (C), chord change rate (D), average loudness (E), RMS (F), beats per minute (G) and danceability (H) between the original and reverse versions of musical pieces.**

2)	The rational for the 'unfamiliar control' is still not clear. First, the authors explain that reverse versions still follow tonal harmonic structures, while then these are presented as unfamiliar in their progression.

**Response:** Sorry for the confusion. In Experiment 1, the reversal manipulation serves to investigate the impact of harmonic progressions on blink synchronization. In the reverse version, while relationships between consecutive beats remain unchanged, the direction of harmonic progressions are fundamentally reversed. These novel harmonic structures diverged significantly from the prototypical tonal harmonic progressions in the original version, resulting in reduced familiarity for participants, which likely disrupted participants' temporal predictions for the musical pieces. Crucially, the reversal manipulation focuses on the harmonic progressions, with alterations in perceptual familiarity emerging as its primary consequence. We have rephrased this sentence to elucidate the rationale underlying our stimulus manipulation with greater precision:

Line 110-112: “This design ensured that the reverse version served as a control for typical tonal harmonic progressions, in which the novel harmonic progressions were less familiar to the participants.”

Additionally, we have highlighted in other sections that no significant differences existed in critical parameters between conditions beyond the harmonic progressions:

Line 119-120: “No significant difference in liking ratings was found between the two versions…”

Line 662-665: “Therefore, the basic musical contents were equal in both versions, but the harmonic progressions were manipulated in the reverse version which would affect listeners’ expectation or familiarity of the musical pieces (S1 Fig).”

3)	Line 212 refers to the "unfamiliar harmonic progressions" requiring higher musical ability to track beat structures - but earlier in the manuscript, the authors claimed that the reversing did not influence perception.

**Response:** We apologize for the misunderstanding. The interpretation mentioned here is based on the results of neural entrainment, revealing a positive correlation between neural entrainment to musical beats and the MET rhythm score (as shown in S3C Fig). To preclude potential misinterpretation, we have incorporated caveats to qualify the interpretation:

Line 217-218: “…higher musical ability may be required to effectively track beat structures in the reverse version for neural entrainment…”

4)	The material construction requires some further explanations. Line 347 indicates the "new tone sequences maintaining the temporal structures of the original musical pieces but lacking pitch information or melodic cues", the figure suggests some adaptations, notably that two different tones were used (voice and bass line) and multiple tones were replaced by one.

**Response:** Sorry for the confusion. In the original musical scores of four-part chorales, the four voice parts are soprano, alto, tenor, and bass. To maintain notational consistency across two stimuli types, both the upper two (soprano and alto) and lower two (tenor and bass) voice parts are annotated with distinct note symbols despite representing acoustically identical pitches (440 Hz). Furthermore, in this polyphonic configuration where all four voice parts converge on acoustically identical pitches at the same beat, the notational representation coalesces into a single visual entity (one tone) per beat position. We have added the tone frequency in Fig 5A and provided detailed descriptions for material construction in the Methods section:

Line 666-668: “In Experiment 2, the original pieces were selected from eight of the ten musical pieces used in Experiment 1, and the tone sequences consisted of frequent standard tones (440 Hz) with timing structures identical to the original pieces.”

5)	- Line 660 (Exp3): Were the deviants always included in the same two pieces which "are interspersed within the block"? if yes, the authors need to acknowledge that participants might learn where the deviant occurs (in the same two pieces) and just listen attentively to those but not all the others. this would considerably change the interpretation of the involved processes - and needs to be discussed in the manuscript.

**Response:** We acknowledge potential ambiguity in interpreting the reviewer's concern. We think the reviewer’s question pertains to the possibility that if the timbre deviants are always included in the same two pieces, the design could lead to implicit learning effects, potentially influencing attentional allocation in Experiment 2.

Our paradigm incorporates some methodological constraints preventing such learning: Timbre deviants were indeed restricted to two specific pieces. However, each piece was presented only once per stimuli type and tempo. Moreover, both the number and inserted positions of timbre deviants differed across conditions. For non-musician participants, recognizing that timbre deviants occurred only in the same two pieces after single exposure would exceed their capacity, and no participants reported awareness of this after the experiment. Additionally, our instructions required participants to listen carefully to the musical pieces to detect randomly occurring timbre deviants and respond via button press as soon as possible. We have revised the description to enhance methodological clarity in the Methods section:

Line 668-672: “To ensure participants’ attention remained focused, we randomly inserted one to five chords deviating in timbre (guitar) into the remaining two musical pieces depending on the duration of piece (2.33 ± 1.44, mean ± SD). For the same piece, both the number of timbre deviants and their insertion positions varied across different stimuli type and tempo.”

6) line 722: how many on and off-beat targets occurred on average and indicate also SD and min/max

**Response:** Thank you for the suggestion. We have added the description:

Line 735-737: “…each musical piece contained at least one and up to four visual targets per condition, depending on the duration of piece, with an equal number of on-beat and off-beat targets within each piece (2.50 ± 1.05, mean ± SD).”

7)	The varying duration of the different excerpts remains surprising. The analyses control for the length differences used by using zero-padding, however, this does not address the possibility that longer pieces allow for different beat entrainment strengths or processes. The authors could regroup the pieces as a function of duration and/or add duration as a predictor or a covariate.

**Response:** The reviewer raised concerns about potential duration effects on beat entrainment. We do not believe that is the case in our study. First, zero-padding approach served specifically to standardize spectral resolution across stimuli (0.0167 Hz) without altering the fundamental spectral properties, not to ignore duration effects.

Most importantly, we conducted surrogate tests in the spectral domain to derive a null distribution of the modulation spectrum of different musical pieces in one condition, then the modulation spectra can be normalized according to the null distributions within each condition. This ensured that the estimated synchronization strength was not biased by trial length but instead reflected genuine frequency-specific modulation.

In addition, within each experimental condition, the excerpts spanned a comparable range of durations (22.59–59.30 s). Thus, any potential duration effects would apply equally across conditions and are unlikely to explain the observed differences in blink synchronization, neural entrainment, or their correspondence. The varying duration did not influence the results qualitatively since different conditions included musical pieces of the same varying duration.

For these reasons, we did not include excerpt duration as a predictor or covariate in the main analyses. While we acknowledge that stimulus duration could, in principle, modulate entrainment strength, our normalization procedure and the comparable duration ranges across conditions make it unlikely that excerpt length affected our findings. We have clarified this point in the Methods sections:

Line 830-835: “To account for differences in trial length, we conducted surrogate test in the spectral domain to derive a null distribution of the amplitude modulation spectra for each independent variable. We then z-scored the raw amplitude spectra with respect to the surrogate mean, effectively normalizing them to remove biases introduced by varying stimulus durations. Next, we averaged the amplitude spectra across the 10 pieces for each condition.”

Analyses/Results

Lines 697-699: Please add your rational of 6000 Data points for the FFT to share with the reader.

**Response:** Thank you for the suggestion. We have added the rational：

Line 826-830: “The blink data for each trial and participant were transformed into the frequency domain using a FFT with zero-padding of 6000 points. This achieved a consistent spectral resolution of 0.0167 Hz across the conditions, enabling precise quantification of the amplitude modulation spectra at the beat rate.”

Please indicate how many blinks were actually performed during each of the musical pieces, that is the analyses are based on N blinks per trial (specified for each piece with its indicated duration)?

**Response:** We thank the reviewer for this question. Our frequency-domain analyses are based on the temporal structure of the blink time series rather than the absolute number of blinks per trial. Thus, the synchronization strength reflects the periodicity of blinking relative to the beat rather than blink count.

That said, conceptually, the FFT captures the dominant periodicity of the blink time series by transforming event timing into frequency components. Thus, a spectral peak around 1 Hz over a 60-second trial indicates that, on average, blinks occurred once per second, corresponding to approximately 60 blinks. While FFT analysis focuses on periodic structure rather than absolute counts, this relationship illustrates how frequency-domain estimates relate to the number of blinks observed over the trial. Importantly, our analyses rely on the periodic structure of blinks rather than raw counts, and the surrogate-normalized FFT ensures that synchronization strength is independent of absolute blink numbers or trial length.

Line 350 "while their eye movements were recorded"? only blinks were considered?

**Response:** Yes, only blinks were considered in our study. We have rephrased the sentence to ensure the consistency with the preceding methodological descriptions:

Line 356: “…while their eye-tracking data were recorded.”

Results/Discussion etc.

-	The lateralisation of the results should be further explained and integrated in the literature - why left- and not right-lateralized or bilateral? Furthermore, line 325 indicates that "better blink and neural synchronisation may be associated with lower neurone density in the left pSLF and AF". The question comes up whether it is about left-lateralisation or rather about the ratio of the involved structures across the two hemispheres and the "lower" indicates a benefit of enhanced asymmetry, thus requiring the inclusion of the right hemisphere to distinguish between these options?

**Response:** Thank you for raising this concern. In our analyses, we concurrently examined four bilateral white matter tracts as well as their corresponding lateralization indices (LI) in relation to blink and neural synchronization. The results demonstrated that only the neurite density index (NDI) of the left pSLF and AF exhibited significant associations with blink and neural synchronization measures. This indicates that the effect is not driven by overall hemispheric balance or asymmetry, but rather reflects a specific contribution of the left pSLF and AF. In particular, we interpret the lower NDI as indicating greater neural efficiency, possibly associated with synaptic pruning, which in turn support more efficient auditory-motor processing of musical beats.

We have expanded the relavant sentence to further discuss the results:

Line 522-526: “Consistent with these findings, our results suggest that lower NDI in the left pSLF and AF supports higher auditory-motor synchrony ability. This may reflect greater neural efficiency from synaptic pruning within these left-hemisphere tracts, rather than hemispheric asymmetry per se, thereby underscoring their unique role in the effective processing of musical beats.”

-	Line 443: The authors conclude that "blink synchronisation is a spontaneous behaviour induced by active engagement in music listening, rather than a response to passive auditory stimulus". However, the authors need also to consider the possibility that the visual task itself interferes with the eye-movements necessary for the task - and it is not related to passive versus active listening. - this would need to be acknowledged throughout the manuscript. The authors integrate it around line 555, but need to temper it elsewhere in the manuscript.

**Response:** Thank you for pointing this out. We have rephrased the sentence as:

Line 448-450: “A plausible explanation is that blink synchronization is modulated by task relevance and the allocation of auditory attention.”

-	The result with faster off-beat responses in the visual modality contradicts previous findings with better visual task performance for on-beat positions (e.g., Ecoffier et al., 2010; Trost et al., 2014; Bolger et al., 2013*) - the authors discuss potential differences with experimental paradigms (lines 555 etc), but it is not clear why these differences should actually matter and/or which underlying mechanisms involved are disturbed etc. Please clarify.

**Response:** Thank you for the valuable suggestion. We have expanded the paragraph to further clarify the underlying mechanisms as follows:

Line 574-588: “This disparity is likely attributable to several factors. First, most studies supporting rhythmic effects employed simple geometric shapes or objects as stimuli, with tasks requiring the detection or discrimination of low-level perceptual features, unlike the pseudowords used in studies yielding null findings in which required lexical-semantic integration, introducing competitive resource allocation that may override rhythmic effects. Second, the temporal relationship between beats and off-beat targets significantly influences outcomes. While off-beat targets in many prior studies appeared at fixed intervals with respect to the beat, in both the current study and previous studies reporting null-effects, off-beat targets were randomly distributed relative to the beat and may impair the precision of temporal prediction. Finally, methodological differences in experimental design may have contributed to the inconsistent findings. Studies reporting rhythmic effects typically used blocked designs, whereas our study employed an intermixed design in which on-beat and off-beat targets occurred within the same block. This design likely weakened the temporal expectations induced by the musical rhythm, thereby diminishing the rhythmic effect on target detection.”

-	Line 582: the observation that eye blinks are more likely to occur *between* two beats contradicts the claims throughout the manuscript that eye blinks "synchronise" to the beat.

**Response:** Thank you for raising this point. However, the observation that eye blinks were more likely to occur between musical beats was specific to Experiment 4, in which blink synchronization disappeared. We have modified the sentence to clarify this point:

Line 591-593: “As illustrated in S5 Fig, eye blinks were more likely to occur between two beats during the visual detection task in Experiment 4, potentially facilitating the perceptual processing of off-beat targets.”

Other points:

Wording is imprecise in several places, for example line 294 "modulated by cognitive factors, such as repetition and reversal manipulations" These later two refer to implementations and manipulations of the experimenter rather than cognitive factors or processes of the participants.

**Response:** Thank you for the thoughtful suggestion. We have replaced “cognitive factors” with “experimental factors”:

Line 298-300: “…the blink-neural relation is modulated by experimental factors such as repetition and reversal manipulations.”

Idem line 393, it seems a stretch to refer to a deviant detection task as 'cognitive performance" - perceptual task?

**Response:** We apologize for this confusion. Experiment 3 specifically examined whether the amplitude of blink synchronization predicts behavioral performance in the detection task, rather than merely associating with the task. We have refined the wording to delineate this point more clearly.

Line 398-399: “In Experiment 3, we investigated whether blink synchronization correlates with behavioural performance in a detection task.”

Line 29: how is "strength" here defined?

**Response:** Here, “strength” refers to “blink amplitude”.

Line 515: lower neuron density - left or right hemisphere?

**Response:** In those studies, lower neurite density was observed at the whole-brain level rather than in a specific hemisphere. We have clarified this in the manuscript:

Line 519-522: “A series of studies have similarly reported that lower neurite density in white and gray matter at the whole-brain level is linked to higher intelligence, better reading and phonological skills, and improved cognitive performance [57-59].”
