# Supplementary material for: Eye blinks synchronize with musical beats during music listening
Source: PLoS Biol. 2025 Nov 18;23(11):e3003456. doi: 10.1371/journal.pbio.3003456 (PMC12626317; doi:10.1371/journal.pbio.3003456)
Supplement: Attachment — Submitted filename: Response to Reviewers_final.docx [file pbio.3003456.s015.docx]

**Reviewer #1**: I thank the authors for their thorough responses to my comments and suggestions. I just have the following points to be clarified in a revision:

**Response:** Thank you.

- Line 448: yes, "task relevance", but please clarify that in the present case it is the visual task modality itself that might interfere with eye-movements necessary for the task and not related to passive/active listening.

**Response:** Thank you for your advice. We have clarified this in the revised manuscript as follows:

Line 451-454: “A plausible explanation is that blink synchronization is modulated by task modality and the allocation of auditory attention, in which the visual task itself may interfere with the eye blinks and also divert attention from the auditory stream.”

- I understand the authors' analyses in the frequency-domain and that the focus is about the periodicity of blinking relative to the beat rather than blink count, thus leading to the FFT analyses. However, the report about how many blinks were performed per trial (or across trials, taking in consideration the duration) is still relevant as it provides the reader with the information about how many sample points are used to calculate the periodicity. Ideally, 1Hz corresponds to approximately 60 blinks, but you should tell readers the real numbers (how many blinks) that are used to calculate your periodicity. This could be done in a supplementary table listing both calculated periodicity Hz and number of sample points used to get this estimation.

**Response:** Thank you for your suggestion. We have added the blink rate (blinks/min) for each condition in the Methods section and Supporting Information (see S3 Table):

Line 773-774: “…were removed (see S3 Table for the mean and SD of the blink rate for each condition).”

**S3 Table. The mean and standard deviation of the blink rate for each condition after preprocessing.** Values are mean ± SD (units: blinks/min).

| **Experiment 1** | | | | |
| --- | --- | --- | --- | --- |
|  | Repetition 1  (1.416 Hz) | Repetition 2  (1.416 Hz) | Repetition 3  (1.416 Hz) | |
| Original | 19.99 ± 10.37 | 20.02 ± 10.98 | 19.99 ± 11.24 | |
| Reverse | 19.21 ± 9.59 | 19.00 ± 9.75 | 19.17± 9.33 | |
| **Experiment 2** | | | | |
|  | 66 bpm  (1.100 Hz) | 85 bpm  (1.416 Hz) | 120 bpm  (2 Hz) |  |
| Original pieces | 19.76 ± 13.47 | 20.58 ± 15.74 | 19.37 ± 13.73 |  |
| Tone sequences | 18.73 ± 12.92 | 19.96 ± 13.63 | 18.42 ± 13.16 |  |
| Baseline | 13.43 ± 11.50 | | |  |
| **Experiment 3** | | | |  |
|  | 85 bpm  (1.416 Hz) | | | |
| Auditory target detection | 21.32 ± 12.88 | | |  |
| **Experiment 4** | | | |  |
|  | 85 bpm  (1.416 Hz) | | | |
| Visual target detection | 15.18 ± 9.59 | | |  |

Again, it should be noted that the dominant periodicity of the blink time series reflects temporal alignment with the musical beat rather than a continuous oscillation. Blinks may occur around the beat over certain intervals, creating a spectral peak at the beat frequency despite their sparse distribution. By contrast, the blink rate of ~20 blinks/min reflects a lower overall count, as participants did not blink on every beat. In other words, the dominant frequency of blink occurrence can approximate the beat frequency over specific periods, although the signal may not be continuously oscillatory throughout the entire recording period. We have further clarified this in the Methods section:

Line 774-778: “Within each trial, data were represented as binary strings, with values set to 1 during blink occurrences and 0 otherwise. The inclusion of blink duration yields more nuanced temporal information compared to blink rate quantification, enabling a more comprehensive assessment of the dominant periodicity of temporal structure in the inherently discrete blink time series.”
